# CHOP-mediated Gasdermin E expression promotes pyroptosis, inflammation, and mitochondrial damage in renal ischemia-reperfusion injury

**DOI:** 10.1038/s41419-024-06525-9

**Published:** 2024-02-22

**Authors:** Nannan Ma, Hao Lu, Ning Li, Weijian Ni, Wenbo Zhang, Qiang Liu, Wenzheng Wu, Shichao Xia, Jiagen Wen, Tao Zhang

**Affiliations:** 1 Department of Urology, The Second Affiliated Hospital of Anhui Medical University, Hefei, Anhui People’s Republic of China; 2 Inflammation and Immune Mediated Diseases Laboratory of Anhui Province, Anhui Institute of Innovative Drugs, School of Pharmacy, Anhui Medical University, Hefei, Anhui People’s Republic of China; 3 Department of Nephropathy, The Zhongda Affilicated Hospital of Southeast University, Nanjing, Jiangsu People’s Republic of China; 4 Department of Pharmacy, Centre for Leading Medicine and Advanced Technologies of IHM, Anhui Provincial Hospital, The First Affiliated Hospital of USTC, Division of Life Sciences and Medicine, University of Science and Technology of China, Hefei, Anhui People’s Republic of China; 5 Anhui Provincial Key Laboratory of Precision Pharmaceutical Preparations and Clinical Pharmacy, Hefei, Anhui People’s Republic of China

**Keywords:** Acute kidney injury, Molecular biology

## Abstract

In clinical practice, renal ischemia-reperfusion injury (IRI) is a common cause of acute kidney injury (AKI), often leading to acute renal failure or end-stage renal disease (ESRD). The current understanding of renal IRI mechanisms remains unclear, and effective therapeutic strategies and clear targets are lacking. Therefore, the need to find explicit and effective ways to reduce renal IRI remains a scientific challenge. The current study explored pyroptosis, a type of inflammation-regulated programmed cell death, and the role of Gasdermins E (GSDME)-mediated pyroptosis, mitochondrial damage, and inflammation in renal IRI. The analysis of human samples showed that the expression levels of GSDME in normal human renal tissues were higher than those of GSDMD. Moreover, our study demonstrated that GSDME played an important role in mediating pyroptosis, inflammation, and mitochondrial damage in renal IRI. Subsequently, GSDME-N accumulated in the mitochondrial membrane, leading to mitochondrial damage and activation of caspase3, which generated a feed-forward loop of self-amplification injury. However, GSDME knockout resulted in the amelioration of renal IRI. Moreover, the current study found that the transcription factor CHOP was activated much earlier in renal IRI. Inhibition of BCL-2 by CHOP leaded to casapse3 activation, resulting in mitochondrial damage and apoptosis; not only that, but CHOP positively regulated GSDME thereby causing pyroptosis. Therefore, this study explored the transcriptional mechanisms of GSDME during IRI development and the important role of CHOP/Caspase3/GSDME mechanistic axis in regulating pyroptosis in renal IRI. This axis might serve as a potential therapeutic target.

## Introduction

Over the past few years, substantial advancements have been made in the treatment and prevention of AKI, however, it has still a high degree of morbidity and mortality [1]. Each year, more than 13 million people suffer from AKI, which is associated with prolonged hospitalization and progression toward ESRD [2, 3]. Furthermore, its sequelae lead to several consequences, including high consumption of medical resources [4], significant economic burden on the health care system [5], and enormous pressure on chronic renal replacement therapy centers [6]. However, this global public health problem has no clear target or effective treatment yet. An important causative factor leading to the development of AKI is IRI. IRI is usually caused by several factors, including hypovolemic shock, infectious shock, partial nephrectomy, and renal transplantation. The pathology of IRI is characterized by the death of renal tubular epithelial cells, which leads to structural destruction and dysfunction. The IRI caused by surgery and other related clinical procedures is an important risk factor for AKI and ESRD and poses a serious threat to human health. Therefore, the molecular mechanism of the renal IRI needs to be investigated to develop novel therapeutic approaches.

Pyroptosis, a form of inflammatory cell death, is characterized by pores formation on the membrane. It is accompanied by the release of inflammatory factors, including IL-1β, IL-18, in response to pathogenic injury [7]. Moreover, the Gasdermins protein family, including GSDMA, B, C, D, and E, plays a vital role in the formation of cell membrane pores. Among them, GSDMD and GSDME are the key proteins involved in the pathogenesis of pyroptosis [8, 9]. The studies conducted in the past decade have proposed two pathways of pyroptosis, namely the classical and the non-classical pathways. The classical pathway is triggered by caspase-1/GSDMD in response to various infections and immunological challenges [10], while the non-classical pathway is triggered by the activation of caspase-11/4/5/GSDMD following the recognition of cytoplasmic lipopolysaccharide (LPS) [11, 12]. A recent study has shown that pyroptosis is linked to the development of AKI, triggering inflammatory injury and facilitating the progression from AKI to chronic kidney disease (CKD) in a life-threatening manner [13–15]. The single-cell sequencing analysis of renal IRI at different periods showed a crucial role of pyroptosis in the IRI [16]. The GSDMD-mediated pyroptosis has been extensively investigated in AKI, it was observed that GSDMD showed high expression in whole renal cell lysates but not in isolated renal tubular epithelial cells in the cis-induced AKI [17]. LPS-stimulated Nlrp3^CA/+^/GSDMD-deficient mice continued to secrete IL-1β and IL-18, indicating that the activation of inflammatory factors was not associated with GSDMD. However, the remedial inflammatory pathway, involving caspase-8/-3-GSDME, was activated [18]. Moreover, GSDME-mediated pyroptosis serves as the initiation point of renal tubular injury in ureteral obstruction, which can lead to the late progression of hydronephrosis, inflammation, and fibrosis [19]. GSDME activation and high expression by chemotherapeutic drugs can lead to caspase-3 cleavage of it. This transformation between apoptosis and pyroptosis could be employed as a therapeutic strategy for tumors [20]. These findings confirmed the important and stabilizing role of GSDME in the occurrence of pyroptosis; however, the detailed mechanisms involved in renal IRI are still unknown.

The endoplasmic reticulum (ER) coordinates the processing, folding, and transportation of intracellular proteins to ensure their quality [21]. Exposure to unfavorable stimuli from the external environment or conditions, such as genetic mutations, hypoxia, nutritional deficiencies, and oxidative stress, can induce ER stress (ERS), leading to the accumulation of unfolded and misfolded proteins in the lumen, which activates the unfolded protein response (UPR) as a defense mechanism against unfavorable external environmental conditions. Under severe and sustained ERS conditions, thereby pointing subsequently to multi-pathway cell death by the transcription factor CHOP [22]. CHOP is activated as a transcription factor, which inhibits the anti-apoptotic protein BCL-2, leading to apoptosis [23]. Moreover, CHOP can regulate the conversion of multiple forms of cell death in different diseases. For example, it can convert apoptosis to ferroptosis, autophagy, and pyroptosis in liver diseases [24]. A recent study confirmed multiple forms of cell death, including apoptosis, pyroptosis, necroptosis, and ferroptosis in renal IRI [16]. However, which the mechanisms through CHOP regulates pyroptosis in renal IRI have not been studied yet.

The current study showed that GSDME was upregulated in renal IRI, which mediated pyroptosis, leading to mitochondrial damage and the release of inflammatory factors. The transcription factor CHOP inhibited BCL-2, leading to apoptosis. Moreover, CHOP directly activated GSDME, which played a central role in the regulation of apoptosis and pyroptosis in renal tubular epithelial cells. Collectively, the findings of the current study elucidated the important role of the CHOP/Caspase3/GSDME mechanistic axis in regulating apoptosis and pyroptosis in renal IRI, which might serve as a potential therapeutic target.

## Results

### Renal ischemia-reperfusion induced pyroptosis, inflammation, and mitochondrial damage in mice

**Fig. 1:**
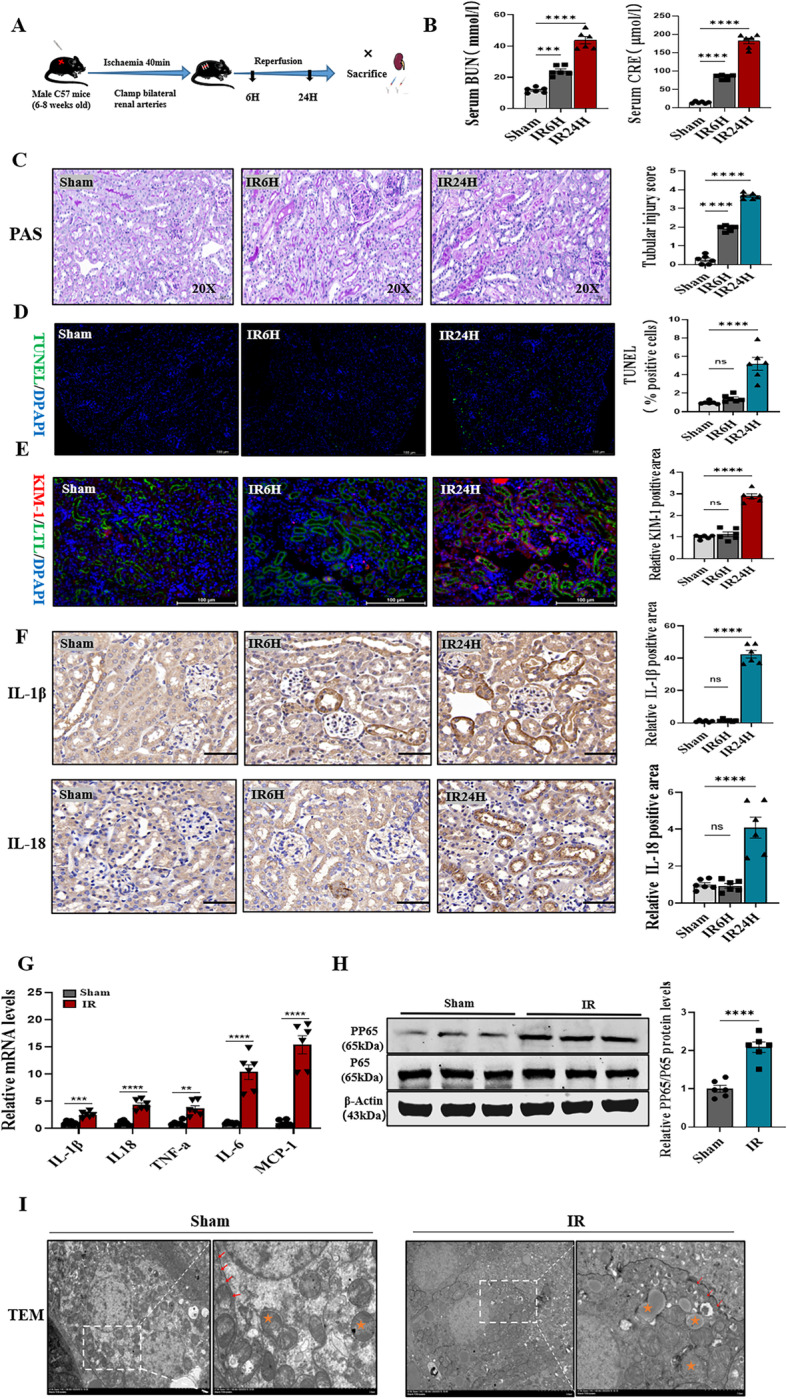
Renal ischemia-reperfusion induced pyroptosis, inflammation and mitochondrial damage in mice. **A** C57 male mice was clamped bilateral renal arteries for 40 min, reperfusion for 6 and 24 h, and then sacrificed under anesthesia. **B** BUN and Scr levels were significantly increased in IR6H and IR24H compared with Sham. *n* = 6/group. **C** PAS-stained histopathological assessment of IR6H and IR24H mice revealed increased tubular damage in comparison to Sham, quantified by tubular damage scores. *n* = 6/group, Scale bar: 50 μm. **D** Representative micrographs show terminal deoxynucleotidyl transferase–mediated dUTP nick end-labeling (TUNEL)-positive cells in different groups, *n* = 6/group, Scale bar: 100 μm. **E** Representative immunofluorescence image of KIM-1 and LTL were expressed in different groups of mice kidney. Red fluorescence represents KIM1 and green fluorescence represents LTL. The protein fluorescence intensity/area between the two groups are shown, *n* = 6/group Scale bar: 100 μm. **F** Representative immunohistochemical staining of IL-1β, IL-18 in IRI mice kidney, compared with Sham. **G** IL-1β, IL-18, TNF-a, IL-6, MCP-1 mRNA were determined by western blotting and qPCR, respectively and (**H**) PP65/P65 protein, in mice kidney. *n* = 6/group. **I** Representative TEM of mitochondria and cell membrane in renal tissue of experimental mouse groups, TEM analysis showed obvious mitochondrial and cell membrane morphological changes in renal tubular epithelial cell. Yellow stars represent mitochondria, Red arrows point to the cell membrane. *n* = 6/group Scale bar: 5 μm and Scale bar: 2 μm (magnification). For all panels, *p* value was determined by unpaired two-tailed Student’s *t* test or one-way ANOVA with Bonferroni post hoc test for multiple comparisons. Data are expressed as mean ± SEM. Quantification on the blots derive from samples of the same experiment and gels/blots were processed in parallel. ns not significant *p* > 0.05, *p < 0.05, **p < 0.01, ***p < 0.001, ****p < 0.0001.

The 6-h and 24-h mouse IRI models were established to investigate the mechanisms of injury (Fig. 1A). It was found that at 6 h, serum creatinine and urea nitrogen levels were elevated and reached even higher levels at 24 h (Fig. 1B). The Periodic Acid-Schiff (PAS) staining showed signs of tubular damage, including attenuation of tubular dilatation, loss of brush border, tubular cell dropout, and cast formation, at 6 h. The severity of damage was higher at 24 h (Fig. 1C). Moreover, TUNEL experiments showed cell death at 6 h, which was more significant at 24 h (Fig. 1D). The immunofluorescence, transcriptional analysis, and immunoblotting of KIM-1, a marker of tubular epithelial cell damage, showed significant elevation at 24 h (Fig. 1E and Supplementary Fig. 1A). Inflammatory factors IL-18 and IL-1β are marker of pyroptosis. Immunohistochemistry analysis showed that IL-18 and IL-1β were significantly elevated at the 24-h time point (Fig. 1F). Meanwhile, F4/80+ were significantly expressed in renal tubular epithelial cells followed by inflammatory cell invasion in IRI of 24-h (Supplementary Fig. 1B). Therefore the 24-h renal IRI model was selected for the subsequent experiments. The mRNA showed that the expression levels of inflammatory factors, including IL-1β, IL-18, TNF-a, IL-6, and MCP-1 increased in renal IRI (Fig. 1G). Similarly, immunoblotting showed that PP65 was highly expressed in IRI (Fig. 1H). Transmission electron microscopy was performed on mouse kidney tissues to further confirm the damage manifestation of cell death and the subcellular organelle alteration following IRI. The results showed discontinuous, broken, and porous cell membranes of renal tubular epithelial cells. Moreover, mitochondria were shrunken with ruptured membranes and the mitochondrial cristae were swollen or absent (Fig. 1I). These evidences confirmed that pyroptosis, inflammation, and mitochondrial damage occurred in mouse renal IRI.

### Gasdermin E, but not GSDMD, was activated in mouse renal IRI

**Fig. 2:**
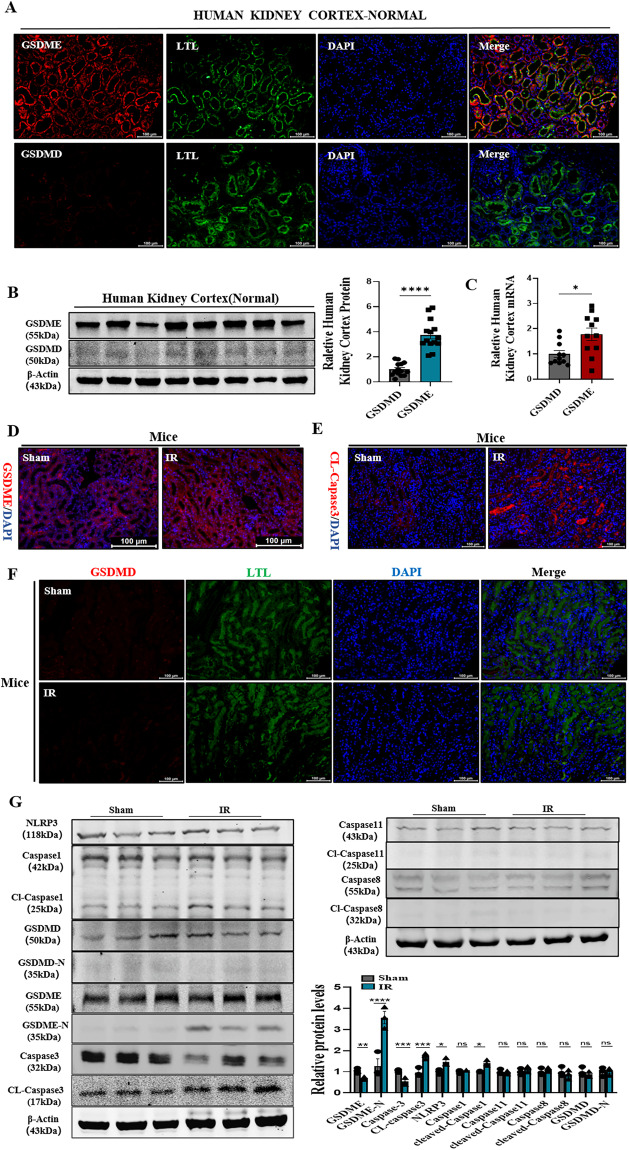
Gasdermin E, but not GSDMD, was activated in mouse renal IRI. **A** Representative immunofluorescence image of GSDME and GSDMD in human renal cortex (Normal). Red fluorescence represents GSDME and GSDMD, and Green fluorescence represents Lotus Tetragonolobus Lectin (LTL), proximal tubular epithelial cell marker. *n* = 6/group Norma Scale bar: 100 μm; Scale bar: 20 μm(magnification). **B** Western blotting analysis of GSDME and GSDMD expression in human renal cortex (Normal). *n* = 14/group. **C** Quantitative polymerase chain reaction (qPCR) analysis of the gene expression relative to GSDMD as well as GSDME in human renal cortex (Normal). *n* = 14/group. **D**, **E** Representative immunofluorescence image of GSDME and cleaved-caspase3 were expressed in mice kidney. Red fluorescence represents GSDME and cleaved-caspase3. **F** Representative immunofluorescence image of GSDMD in mice kidney, Red fluorescence represents GSDMD. *n* = 6/group Scale bar: 100 μm. **G** Western blotting analysis of GSDME, GSDME-N, Caspase3, Cleaved-Caspase3, NLRP3, Caspase1, Cleaved-Caspase1, Caspase8, Cleaved-Caspase8, Caspase11, Cleaved-Caspase11, GSDMD, GSDMD-N expression in mice kidney. *n* = 6/group. For all panels, *p* value was determined by unpaired two-tailed Student’s *t* test or one-way ANOVA with Bonferroni post hoc test for multiple comparisons. Data are expressed as mean ± SEM. Quantification on the blots derive from samples of the same experiment and gels/blots were processed in parallel. ns not significant *p* > 0.05, *p < 0.05, **p < 0.01, ***p < 0.001, ****p < 0.0001.

GSDMD and GSDME are the key proteins involved in the pathogenesis of pyroptosis. Their roles in inducing pyroptosis, a dominant factor in renal IRI, were investigated. A total of 14 normal human renal specimens (7 males and 7 females) were collected from the patients who underwent renal cell carcinoma radical surgery to evaluate the basal expression levels of GSDMD and GSDME in renal tubular epithelial cells. The expression levels were observed using immunofluorescence staining. The results showed that in human proximal renal tubules, GSDME expression was significantly high (Fig. 2A), while that of GSDMD was low. The transcriptional analysis and immunoblotting were used to further confirm their expression levels in human proximal renal tubules. The mRNA and protein expression levels of GSDME were high, while those of GSDMD were low (Fig. 2B, C). Notably, immunofluorescence showed that the expression levels of GSDME and cleaved-caspase3 increased (Fig. 2D, E), however, GSDMD was barely expressed on renal tubular epithelial cells following the mice renal IRI (Fig. 2F). Moreover, the expression of each inflammatory caspase was compared using immunoblotting analysis. The results showed that the levels of cleaved caspase3 and GSDME-N were significantly higher in IRI, comparing NLRP3, caspase1 and GSDMD. Similarly, caspase11 and caspase8 expression levels were not significantly different between the sham and IRI groups (Fig. 2G). Interestingly, we found that GSDMD was predominantly expressed in the renal medulla (Supplementary Fig. 1C). In this process, Gasdermin E and caspase3 were activated, but not GSDMD, in proximal renal tubular epithelial cells. These results confirmed that GSDME was highly expressed in human and mouse renal tubular epithelial cells, which might have a vital role in renal IRI.

### Gasdermin E knockout attenuated the renal IRI of pyroptosis, inflammation, and mitochondrial damage

**Fig. 3:**
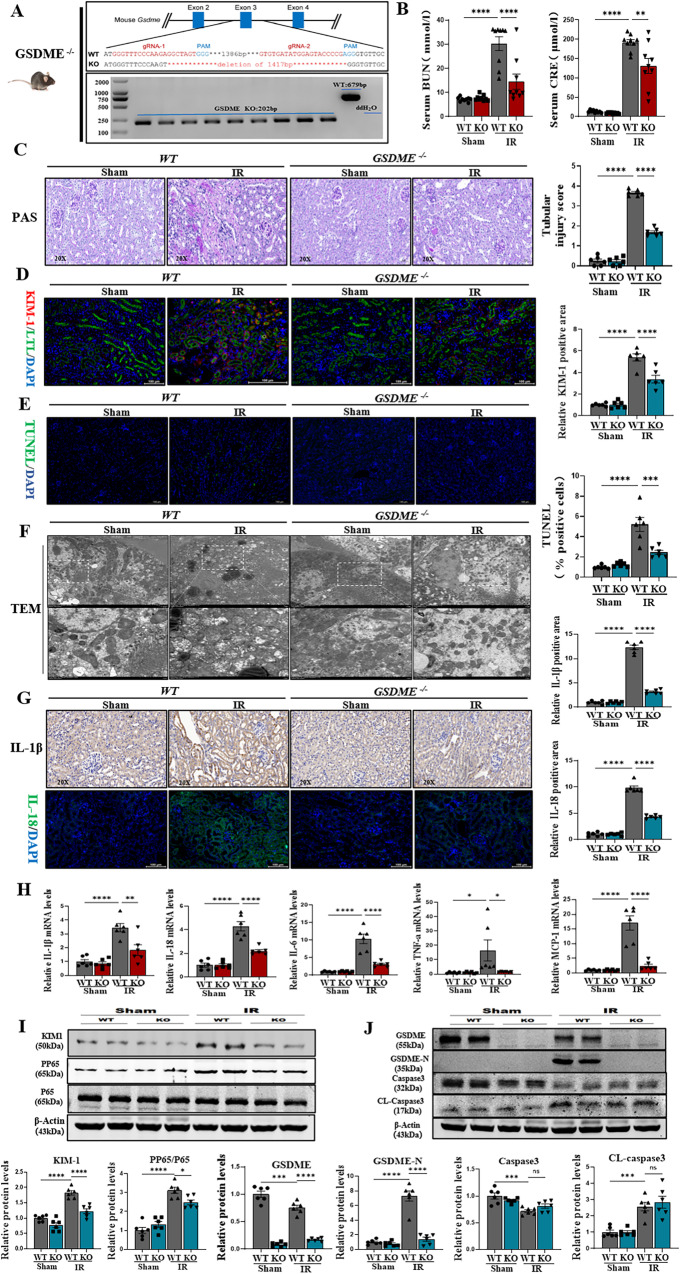
Gasdermin E knockout attenuated the renal IRI of pyroptosis, inflammation, and mitochondrial damage. **A** Successful GSDME knockout confirmed using mouse tail genotyping. **B** BUN and Scr levels were significantly decreased in GSDME^−/−^ mice compared with Sham, *n* = 6/group. **C** PAS-stained histopathological assessment of GSDME^−/−^ mice showed reduced renal tubular injury compared to IRI mice, quantified by tubular damage scores, *n* = 6/group, Scale bar: 50 μm. **D** Representative immunofluorescence image of KIM-1 and LTL were expressed in mice kidney. GSDME^−/−^ decreased the expression of KIM1 protein. Red fluorescence represents KIM1 and green fluorescence represents LTL. The protein fluorescence intensity/area in each group were shown, *n* = 6/group, Scale bar: 100 μm. **E** Representative micrographs show terminal deoxynucleotidyl transferase-mediated dUTP nick end-labeling (TUNEL)-positive cells in different groups, *n* = 6/group, Scale bar: 100 μm. **F** Representative TEM of mitochondria in renal tissue of experimental mouse groups, TEM analysis showed obvious mitochondrial morphological changes in different groups. *n* = 6/group, Scale bar: 5 μm and Scale bar: 2 μm (magnification). **G** Representative immunofluorescence and immunohistochemistry (IHC) image of IL-18, IL-1β were expressed in different groups, green fluorescence represents IL-18, the protein fluorescence intensity/area in each group are shown, *n* = 6/group, Scale bar: 50 μm; Scale bar: 100 μm. **H** Real-time PCR detected mRNA levels of IL-1β, IL-18, IL-6, TNF-a, MCP-1 in different groups. **I**, **J** Western blot analysis showed protein expression of KIM1, PP65, GSDME, GSDME-N, Caspase3, Cleaved-Caspase3. For all panels, *p* value was determined by one-way ANOVA with Bonferroni post hoc test for multiple comparisons. Data are expressed as mean ± SEM. Quantification on the blots derive from samples of the same experiment and gels/blots were processed in parallel. ns not significant *p* > 0.05, *p < 0.05, **p < 0.01, ***p < 0.001, ****p < 0.0001.

Subsequently, to explore whether GSDME has a function in the renal IRI. The GSDME knockout mouse was constructed and confirmed by genotyping (Fig. 3A). A significant recovery of renal function was observed in GSDME knockout mice after treating IRI, which was characterized by an obvious reduction in serum creatinine and blood urea nitrogen levels (Fig. 3B). PAS-staining revealed significant renal tubular dilatation, loss of brush border, detachment of tubular cells, and casting formation in the WT model group as compared to those in the GSDME-knockout mice (Fig. 3C). The immunofluorescence and immunoblotting showed decreased expression of KIM1 in GSDME knockout mice (Fig. 3D, I). The renal cell death was also investigated by staining TUNEL+ cells in mice kidneys. It was found that renal cell death improved in the renal tissues of GSDME-knockout mice (Fig. 3E). The analysis of renal tissues using transmission electron microscopy revealed that GSDME-knockout mice maintained the basic morphology of mitochondria, which was characterized by no obvious rupture of vacuoles and preservation of mitochondrial cristae. These results indicated that GSDME-knockout could reverse mitochondrial damage in renal IRI (Fig. 3F). Subsequently, the inflammatory factors, such as IL-1β and IL-18, were both detected by immunohistochemistry and immunofluorescence. It was found that the GSDME knockout decreased the release of IL-1β and IL-18 as well as the expression of F4/80+ (Fig. 3G and Supplementary Fig. 1D). Moreover, the mRNA expression levels of IL-1β, IL-18, IL-6, TNF-a, and MCP-1, also decreased significantly (Fig. 3H). The immunoblotting revealed the expression of PP65 was reduced in GSDME knockout mice (Fig. 3I). These evidences suggested that the GSDME knockout attenuated inflammation. Interestingly, GSDME and GSDME-N were not expressed in the GSDME-knockout mice; however, activated caspase3 still increased (Fig. 3J), suggesting that apoptosis maybe was still present in renal tubular epithelial cells. These results suggest that GSDME had vital role in inducing pyroptosis, inflammation, and mitochondrial damage.

### GSDME-N overexpression promoted pyroptosis and inflammation in renal tubular epithelial cells

**Fig. 4:**
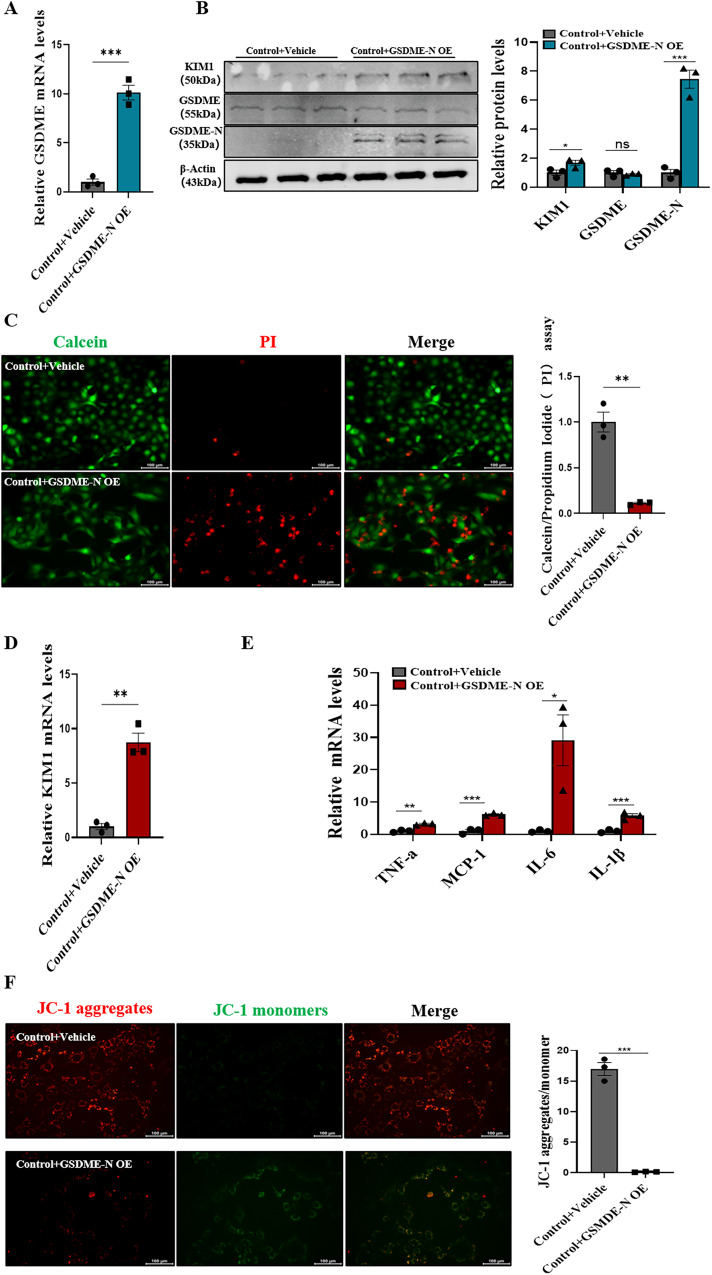
GSDME-N overexpression promoted pyroptosis and inflammation in renal tubular epithelial cells. **A**, **D**, **E** Real-time PCR detected mRNA levels of GSDME, KIM1, TNFa, IL-6, IL-1β between control and GSDME-N overexpression (OE). *n* = 3/group; **B** Western blot analysis showed protein expression of KIM1, GSDME, GSDME-N; **C** Representative Calcein/PI fluorescence stains, Calcein represents living cells, PI represents dead cells, *n* = 3/group, Scale bar: 100 μm. **F** JC-1 staining representation of mitochondrial disability, *n* = 3/group, Scale bar: 100 μm. For all panels, *p* value was determined by unpaired two-tailed Student’s *t* test. Data are expressed as mean ± SEM. Quantification on the blots derive from samples of the same experiment and gels/blots were processed in parallel. ns not significant *p* > 0.05, *p < 0.05, **p < 0.01, ***p < 0.001, ****p < 0.0001.

A previous study identified that caspase3 could cleave GSDME at 267DMPD270 in humans. GSDME-N with pore-forming activity perforated cell membranes to induce pyroptosis in several cell lines [25]. In order to further confirm the effects of GSDME-N-mediated pyroptosis in renal tubular epithelial cells, HK-2 cells were transfected with GSDME-N overexpressing plasmids (residues 1–270aa) in vitro. The immunoblotting showed the expression of GSDME-N was increased in GSDME-N overexpression group (Fig. 4A, B). We detected the damage molecule KIM-1. The results showed that KIM-1 was significantly upregulated in cells overexpressing GSDME-N (Fig. 4B, D). Meanwhile, nuclear PI (Propidium Iodide) staining increased (Fig. 4C). Furthermore, IL-1β, IL-6, TNF-α, and MCP-1 mRNA levels were elevated in cells transfected with GSDME-N-overexpressing plasmid (Fig. 4E). Interestingly we found that mitochondrial damage manifested as a decrease in membrane potential by JC-1 immunofluorescence (Fig. 4F). All these results suggested that GSDME-N directly aggravated human renal tubular cell injury.

### Activated Gasdermin E promoted pyroptosis and induced mitochondrial damage in renal tubular epithelial cells

**Fig. 5:**
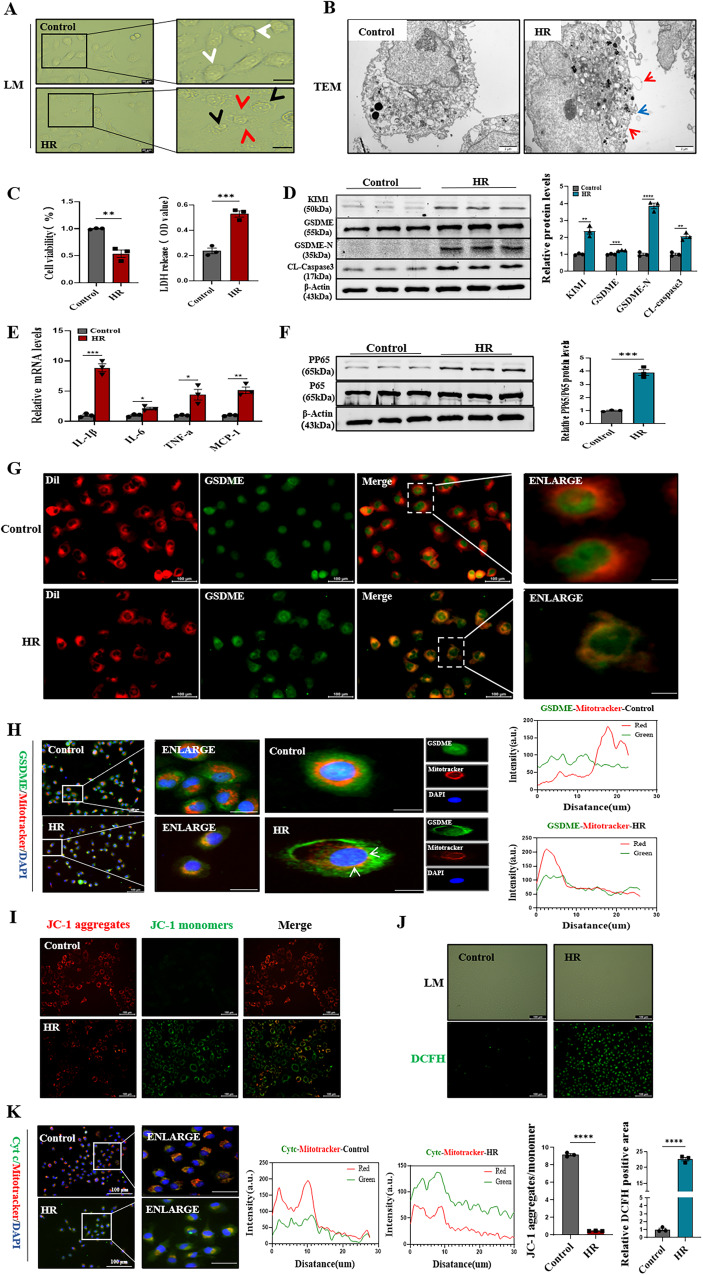
Activated Gasdermin E promoted pyroptosis and localized to mitochondria in renal tubular epithelial cells, which enhanced caspase3 activation and formed a positive feed-forward damage response loop in renal tubular epithelial cells. **A** Representative light microscope images of cells. Red arrows indicate large bubbles from the plasma membrane. White and black arrows represent cells in the normal and model groups, respectively. *n* = 3/group, scale bar: 20 μm. **B** Representative transmission electron microscopy (magnification ×10,000; scale bar = 2 μm) of cells between control and HR. Red arrowheads indicate large bubbles and pores emerging from the plasma membrane. Blue arrowhead indicate spillover organelles. **C** Representative cell viability and LDH release (OD values), respectively. **D** Western blot analysis showed protein expression of KIM1, GSDME, GSDME-N, Cleaved-Caspase3. **E** Real-time PCR detected mRNA levels of IL-1β, IL-6, TNF-a, MCP-1. **F** Western blot analysis showed protein expression of PP65, P65. **G** Representative co-localization images of Dil (labeled in red) and GSDME (labeled in green) in HK-2 cells; Scale bar: 100 μm; Scale bar: 20 μm (magnification). **H** Representative co-localization images of the mitochondrial marker MitoTracker (labeled in red) and GSDME (labeled in green) in HK-2 cells; White arrows indicate mitochondrial and GSMDE co-localization sites. Fluorescence co-localization display showed correlation analysis, *n* = 3/group, Scale bar: 100 μm; Scale bar: 20 μm (magnification). **I** JC-1 staining representation of mitochondrial disability, *n* = 3/group, Scale bar: 100 μm. **J** DCFH staining of ROS level, *n* = 3/group, Scale bar: 100 μm. **K** Representative co-localization images of the mitochondrial marker MitoTracker (labeled in red) and Cytochrome c(cyt-c) (labeled in green) in HK-2 cells; the protein fluorescence intensity/area in each group are shown, *n* = 3/group, Scale bar: 100 μm; Scale bar: 20 μm (magnification). For all panels, *p* value was determined by unpaired two-tailed Student’s *t* test. Data are expressed as mean ± SEM. Quantification on the blots derive from samples of the same experiment and gels/blots were processed in parallel. ns not significant *p* > 0.05, *p < 0.05, **p < 0.01, ***p < 0.001, ****p < 0.0001.

As reported previously, GSDME had little effect on pyroptosis. However, activated GSDME provided similar functions as GSDME-N in inducing pyroptosis. The human renal tubular epithelial cells (HK-2 cells) were used to establish the hypoxia-reoxygenation (HR) model (simulating renal IRI). Electron microscopy revealed that HK-2 cells were swollen and ruptured in HR model, followed by the production of large air bubbles, demonstrating the occurrence of pyroptosis (Fig. 5A). Moreover, transmission electron microscopy suggested that HK-2 cells were swollen, enlarged, and had bubbles in their cell membrane, while the membrane appeared to be broken and organelles spilled out (Fig. 5B). The cell viability decreased, while lactate dehydrogenase (LDH) production increased (Fig. 5C). The immunoblotting and immunofluorescence results showed an increased expression of KIM-1 in HR (Fig. 5D and Supplementary Fig. 2A). Moreover, the expression levels of GSDME, cleaved caspase3, and GSDME-N significantly increased in HR (Fig. 5D). Moreover, immunofluorescence also showed that the cleaved-caspase3 levels increased (Supplementary Fig. 2B). Furthermore, the mRNA expression levels of the inflammatory factors, including IL-1β, IL-6, TNF-a, and MCP-1 (Fig. 5E), as well as the expression of pP65 increased by immunoblotting (Fig. 5F). In combination with Fig. 5B, D, the immunofluorescence double staining in HR showed that the activated GSDME accumulated in membrane, which was signed by Dil (Cell Plasma Membrane Staining Kit) (Fig. 5G). Furthermore, the results also showed the accumulation of activated GSDME in mitochondria (Fig. 5H). Immunofluorescence was performed to confirm mitochondrial damage by decreasing the fluorescence intensity of JC-1 aggregates/monomers and increasing the release of reactive oxygen species (ROS) (Fig. 5I, J). Consistently, cytochrome C was released from mitochondria into the cytoplasm (Fig. 5K). These results suggested that GSDME was cleaved to GSDME-N by activated caspase3 in HK-2 HR models. GSDME-N not only induced pyroptosis but also localized to mitochondria, which caused mitochondrial damage and increased the release of cytochrome C. Notably, cytochrome C could further activate caspase3 [26]. These processes collectively formed a positive feed-forward damage response loop, exacerbating renal tubular epithelial cell pyroptosis.

### CHOP-activated caspase-3 via the mitochondrial apoptosis pathway in renal IRI

**Fig. 6:**
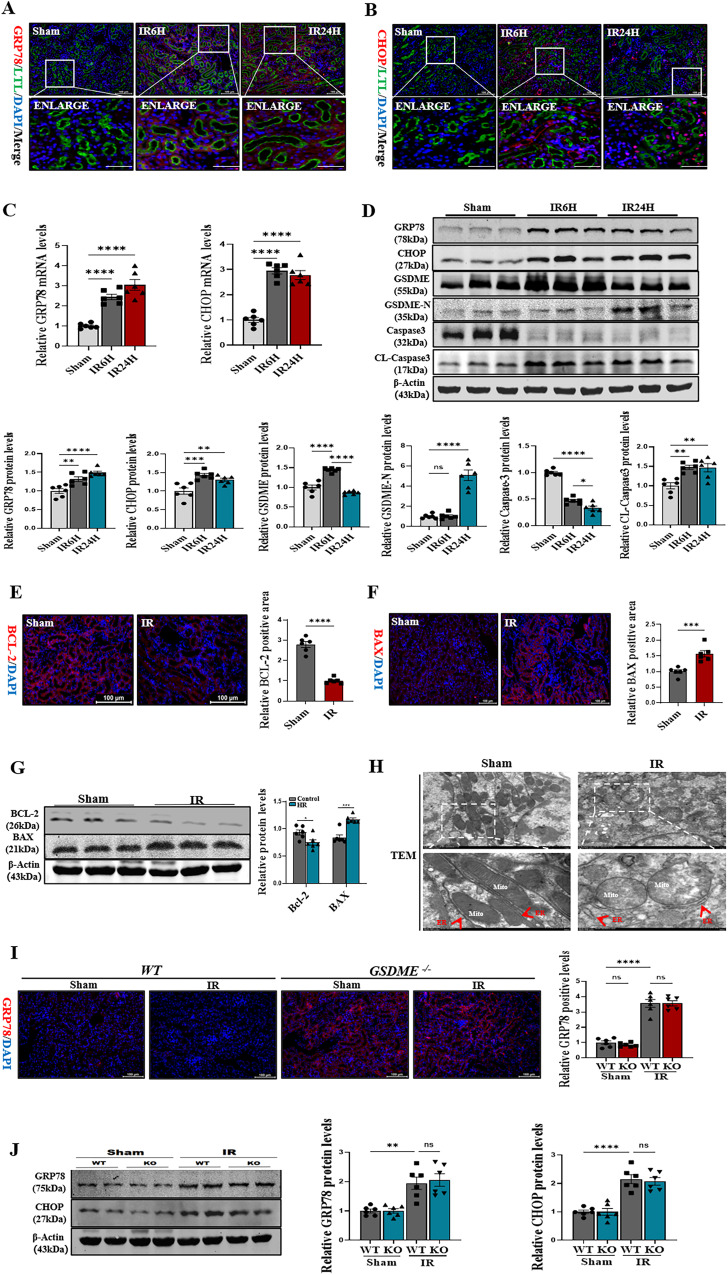
CHOP-activated Caspase-3 occurred apoptosis and pyroptosis in renal IRI. **A**, **B** Representative immunofluorescence image of GRP78 and CHOP in different groups (Sham/IR6H/IR24H). Red fluorescence represents GRP78 and CHOP, respectively. Green fluorescence represents Lotus Tetragonolobus Lectin (LTL). The protein fluorescence intensity/area in each group are shown, *n* = 6/6/6 Sham/IR6H/IR24H, Scale bar: 100 μm; Scale bar: 20 μm (magnification). **C** Real-time PCR detected mRNA levels of GRP78, CHOP in different groups. **D** Western blot analysis showed protein expression of CHOP, GRP78, GSDME, GSDME-N, Caspase3, Cleaved-Caspase3 in different groups. **E**, **F** Representative immunofluorescence image of BCL-2 and BAX in different groups (Sham/IR). Red fluorescence represents BCL-2 and BAX, respectively; the protein fluorescence intensity/area between the two groups are shown, *n* = 6/6 Sham/IR, Scale bar: 100 μm. **G** Western blot analysis showed protein expression of BCL-2, BAX. **H** Representative TEM of mitochondria and endoplasmic reticulum (ER) in renal tissue of experimental mouse groups, TEM analysis showed obvious morphological changes of mitochondria and ER in renal tubular epithelial cells. *n* = 6/group, Scale bar: 5 μm and Scale bar: 2 μm (magnification). **I** Representative immunofluorescence image of GRP78 in different groups. Red fluorescence represents GRP78, the protein fluorescence intensity/area in each group are shown, *n* = 6/6/6/6 Sham: WT/KO; IR: WT/KO, Scale bar: 100 μm. **J** Western blot analysis showed protein expression of CHOP, GRP78. For all panels, *p* value was determined by unpaired two-tailed Student’s *t* test or one-way ANOVA with Bonferroni post hoc test for multiple comparisons. Data are expressed as mean ± SEM. Quantification on the blots derive from samples of the same experiment and gels/blots were processed in parallel. ns not significant *p* > 0.05, *p < 0.05, **p < 0.01, ***p < 0.001, ****p < 0.0001.

Apoptosis and GSDME-mediated pyroptosis share the same upstream regulatory protein caspase3. CHOP, a transcription factor, causes apoptosis by releasing cytochrome C, thereby activating caspase3 through the mitochondrial pathway. This pathway plays an important role in several diseases, including diabetes mellitus, cerebral ischemia, and neurodegenerative diseases [23]. In this study, the activation of caspase3 and regulatory mechanism of GSDME-mediated pyroptosis were investigated. The immunofluorescence double staining in mouse renal IRI revealed that GRP78, an ERS biomarker, and CHOP were highly expressed at 6 and 24 h (Fig. 6A, B). The mRNA and protein levels of GRP78 and CHOP showed similar results (Fig. 6C, D). The immunohistochemistry showed no differential changes in IL-18, IL-1β, and F4/80+ at 6 h, suggesting no inflammation in the early stage of renal IRI (Fig. 1F and Supplementary Fig. 1B). Cleaved-caspase3 and GSDME expression levels were significantly high at 6 h, while no difference in the expression levels of GSDME-N was observed using immunoblotting. However, at 24 h, cleaved-caspase3 showed a sustained high expression, while that of GSDME decreased. A high expression of GSMDE-N was also observed at the 24-h time point (Fig. 6D). The 24-h time point was selected for subsequent experiments because pyroptosis was occurred. The immunofluorescence and immunoblotting showed that BAX was significantly elevated in the model group, while BCL-2 was reduced (Fig. 6E–G). Moreover, transmission electron microscopy of renal tissues revealed mitochondrial damage, ER lumen dilation, and discontinuity of ER structure in renal IRI (Fig. 6H). Notably, GRP78 and CHOP showed high expressions in GSDME-knockout mice; however, GSDME-knockout attenuated the renal IRI (Fig. 6I, J). It was hypothesized that CHOP might be an upstream regulator of GSDME-mediated pyroptosis. Based on the results, it was concluded that the transcription factor CHOP activated caspase-3 through the mitochondrial apoptotic pathway. Elevated GSDME expression with disease progression. Subsequently, activated caspase 3 not only mediated apoptosis but also cleaved GSDME causing pyroptosis, thereby exacerbating renal IRI injury (Fig. 1).

### CHOP knockdown ameliorated mitochondrial-dependent apoptosis in renal tubular epithelial cells

**Fig. 7:**
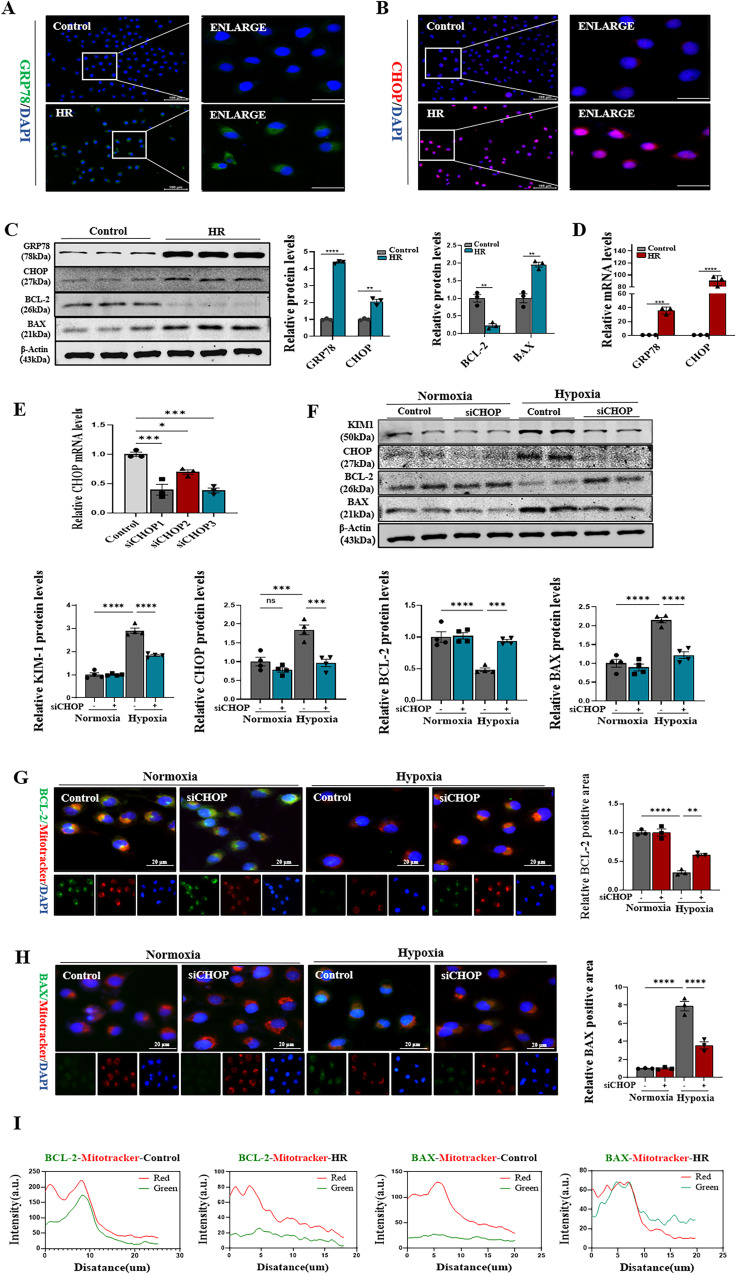
Endoplasmic reticulum stress activated the transcription factor CHOP and CHOP knockdown ameliorated mitochondrial-dependent apoptosis in renal tubular epithelial cells. **A**, **B** Representative immunofluorescence image of GRP78 and CHOP in HK-2 (Control and HR). Green fluorescence represents GRP78; Red fluorescence represents CHOP, the protein fluorescence intensity/area between the two groups are shown, *n* = 3/group, Scale bar: 100 μm, Scale bar: 20 μm (magnification). **C** Western blot analysis showed protein expression of GRP78, CHOP, BCL-2, BAX. **D** Real-time PCR detected mRNA levels of GRP78, CHOP in different groups. **E** Real-time PCR detected mRNA levels of CHOP and CHOP knockdown. **F** Western blot analysis showed protein expression of KIM1, CHOP, BCL-2, BAX in different groups. **G**, **H** Representative co-localization images of the mitochondrial marker MitoTracker (labeled in red) and BCL-2, BAX (labeled in green) in HK-2 cells, the protein fluorescence intensity/area in each group are shown, *n* = 3/group, Scale bar: 20 μm (magnification). **I** BCL-2, BAX and mitochondria fluorescence co-localization display showed correlation analysis in each group, respectively. For all panels, *p* value was determined by unpaired two-tailed Student’s *t* test or one-way ANOVA with Bonferroni post hoc test for multiple comparisons. Data are expressed as mean ± SEM. Quantification on the blots derive from samples of the same experiment and gels/blots were processed in parallel. ns not significant *p* > 0.05, *p < 0.05, **p < 0.01, ***p < 0.001, ****p < 0.0001.

CHOP mediated the mitochondrial apoptotic pathway in renal IRI. The current study investigated mechanism of the transcription factor CHOP in renal tubular epithelial cells. Immunofluorescence showed elevated expression levels of GRP78 and CHOP (Fig. 7A, B), and the mRNA levels of GRP78 and CHOP also significantly increased in HR (Fig. 7D). Moreover, immunoblotting confirmed that the expression levels of GRP78, CHOP, and BAX increased, while that of anti-apoptotic protein BCL-2 decreased (Fig. 7C). To investigate the function of CHOP. Three siRNAs were designed to knock down the transcription factor CHOP. Among them, the third one showed the best results by detecting mRNA decline rates (Fig. 7E). The immunoblotting experiments confirmed that the expression levels of KIM1 and BAX significantly decreased in the CHOP knockdown group, while BCL-2 showed the opposite trend (Fig. 7F). Moreover, immunofluorescence double staining revealed increased expression levels of Bax and decreased expression levels of BCL-2 in mitochondria. However, these trends could be reversed after CHOP knockdown (Fig. 7G, H). Meanwhile, immunofluorescence co-localization and quantitative analysis revealed that BAX and BCL-2 were closely associated with mitochondria in both normal and HR groups (Fig. 7I). Consistent with animal model, immunoblotting revealed that CHOP and cleaved-caspase3 were activated in HR of 3 h, however GSDME-N was high expression in HR of 6 h in renal tubular epithelial cells (Supplementary Fig. 2C). These results suggested that CHOP regulated caspase3 through the mitochondrial apoptotic pathway, which was the key regulator of GSDME-mediated pyroptosis.

### CHOP regulated caspase3/GSDME-mediated pyroptosis, while its knockdown ameliorated pyroptosis in renal tubular epithelial cells

**Fig. 8:**
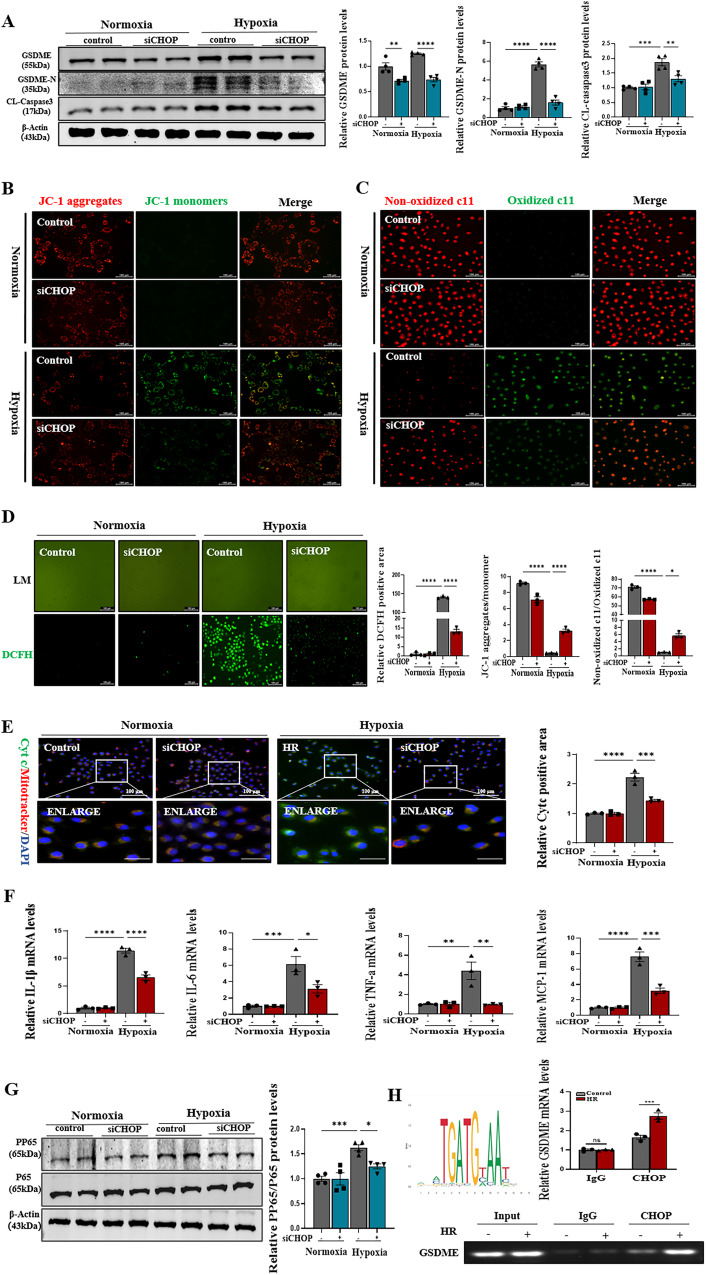
CHOP upregulated GSDME expression, while its knockdown ameliorated pyroptosis, mitochondrial damage, and inflammation in renal tubular epithelial cells. **A** Western blot analysis showed protein expression of GSDME, GSDME-N, Cleaved-Caspase3 in different groups. **B** JC-1 staining representation of mitochondrial disability in different groups, *n* = 3/group, Scale bar: 100 μm. **C**, **D** DCFH staining of ROS and C11 staining of lipid peroxidation levels, which confirmed the release of ROS, *n* = 3/group, Scale bar: 100 μm. **E** Representative co-localization images of the mitochondrial marker MitoTracker (labeled in red) and Cytochrome c(cyt-c) (labeled in green) in HK-2 cells; the protein fluorescence intensity/area in each group are shown, *n* = 3/group, Scale bar: 100 μm; Scale bar: 20 μm (magnification). **F** Real-time PCR detected mRNA levels of IL-1β, IL-6, TNF-a, MCP-1 in different groups. **G** Western blot analysis showed protein expression of P65, PP65 in different groups. **H** Chromatin immunoprecipitation analysis with antibodies against CHOP or IgG, soluble chromatin from PMs, and primers targeting the region spanning the binding site in the GSDME promoter in different groups, *n* = 3/group. For all panels, *p* value was determined by unpaired two-tailed Student’s *t* test or one-way ANOVA with Bonferroni post hoc test for multiple comparisons. Data are expressed as mean ± SEM. Quantification on the blots derive from samples of the same experiment and gels/blots were processed in parallel. ns not significant *p* > 0.05, *p < 0.05, **p < 0.01, ***p < 0.001, ****p < 0.0001.

The high expression of GSDME and activation of caspase3 are one of the necessary conditions for the transition from apoptosis to pyroptosis [20]. The study showed that the expression of CHOP and GSDME mRNA had the same trends (Supplementary Fig. 2D). We hypothesized whether CHOP was involved in the transcription regulation of GSDME. Predictive analysis of the interaction between the GSDME promoter region and CHOP using JASPAR software indicated that CHOP could regulate GSDME (Fig. 8H). Meanwhile, we found that CHOP knockdown decreased the expression of GSDME both in normal and model groups, with a concomitant decrease in GSDME-N and cleaved-caspase3 (Fig. 8A). As the expression of GSDME reduced after the CHOP knockdown, the mitochondrial damage was ameliorated by the reduction of JC-1 aggregates/monomers indices (Fig. 8B), and the release of ROS was decreased, as shown by C11 lipid peroxidation and DCFH staining (Fig. 8C, D). Consistently, the reduction in the release of cytochrome C into the cytoplasm was observed using immunofluorescence double staining after the CHOP knockdown (Fig. 8E). Since GSDME-mediated pyroptosis led to the release of large amounts of inflammatory factors, it was found that the expression levels of PP65 and the mRNA levels of IL-1β, IL-6, TNF-a, and MCP-1 decreased in the CHOP knockdown group (Fig. 8F, G). These studies showed that CHOP was involved in the transcriptional regulation of GSDME by chromatin immunoprecipitation assay, which showed that CHOP interacted with GSDME promoter region AGCTTTCCCTCTTGCACACG (Fig. 8H). These findings indicated the significance of CHOP in the transcriptional regulation of GSDME, which is an important regulator in apoptosis and GSDME-mediated pyroptosis.

## Discussion

Renal IRI is an important causative factor leading to AKI [27]. Over the past few decades, the exploration of renal IRI mechanism has mostly focused on various types of programmed cell death, including apoptosis, necrosis, ferroptosis, and pyroptosis, which have played an indispensable and important role in the explanation of AKI. Among them, pyroptosis is considered to be the most common mode of inflammatory cell death [28]. Previous studies showed that activated inflammatory cysteine asparaginase could mediate pyroptosis, which was characterized by pore formation in the cell membrane and the release of inflammatory factors, including IL-1β [7]. Recent studies on the GSDMs family suggested that both GSDMD and GSDME were the executive proteins of pyroptosis, which played an important role in the development of renal diseases [29]. Typically, GSDMD could be cleaved to GSDMD-N by caspase1, triggering pyroptosis and release of inflammatory factors [30]. GSDMD is known to be involved in the pathogenesis of several diseases, including septic shock, spinal cord injury, and myocardial IRI [31–33]. However, bone marrow-derived macrophages from Nlrp3^CA/+^/Gsdmd^−/−^ mice still secreted IL-1β and IL-18 when challenged with LPS or TNF-α, indicating inflammasome activation independent of GSDMD. A salvage inflammatory pathway, involving caspase-8/-3–GSDME, was activated after NLRP3 activation when the canonical N LRP3-GSDMD signaling was blocked [18]. GSDMD-deficient cells were still susceptible to inflammasome-mediated cell death. Subsequently, inflammasome stimulation of GSDMD-deficient cells led to apoptotic caspase cleavage of GSDME.

The known mutations in the GSDME gene, associated with non-syndromic hearing loss in humans [34], resulted in transcriptional skipping in exon 8, leading to the production of truncated protein with cytotoxic activity [35]. The physiological activation mechanisms of GSDME involve its cleavage by caspase 3 and the formation of pores in the plasma membrane due to GSDME-N fragment oligomerization, leading to cell lysis. GSDME-N also permeabilizes the mitochondrial membrane to augment caspase 3 activation during apoptosis and inflammasome activation [26]. Therefore, GSDME possesses the ability to regulate apoptosis and pyroptosis in response to caspase 3 activators, including chemotherapeutic drugs, tumor necrosis factor, and viral infection [36]. Previous studies reported that GSDME^−/−^ mice were protected from chemotherapy-induced tissue damage and weight loss [37] and proposed that in cancer, the caspase3/GSDME pathway acted as a regulator between apoptosis and pyroptosis [20]. Moreover, GSDME-mediated pyroptosis and inflammation initiate renal tubular injury caused by ureteral obstruction, which can lead to late progression of renal hydronephrosis, inflammation, and fibrosis [19]. The activation of endogenous GSDME can rupture the cell membrane and sustain the release of IL-1β even in GSDMD-sufficient cells. Therefore, blocking GSDME might serve as a potential therapeutic target to treat diseases, such as renal IRI. However, the mechanism of GSDME involvement in renal IRI is not fully understood yet. The current study revealed that GSDME was highly expressed in renal tubular epithelial cells, and GSDME-N not only induced pyroptosis but also localized to mitochondria, which led to mitochondrial damage and the release of cytochrome C. Notably, cytochrome C could further activate caspase3. These processes collectively formed a positive feed-forward damage response loop, exacerbating renal tubular epithelial cell pyroptosis (Fig. 5). GSDME deficiency suppressed pyroptosis-related inflammation and reduced the development of the renal IRI. The protective effects of GSDME deficiency were mediated, at least in part, by inhibiting the local inflammatory response and mitochondrial damage (Fig. 3). The expression levels of GSDME determine the fate of tumor cells in response to the caspase 3 activators. The caspase3/GSDME signal pathway shifted the balance between apoptosis and pyroptosis. At high levels of GSDME, caspase3 could cleave GSDME, triggering pyroptosis. However, the regulatory mechanism involved in GSDME expression is not fully understood yet. It has been reported that p53 can positively regulate the expression levels of GSDME, thereby activating the transcription of many tumor suppressor genes [38]. In atherosclerosis, GSDME might also be activated by STAT3 [25]. Meanwhile, the overactivated METTL3/EVL m6A axis is a potential target for renal fibrosis therapy, which maybe be an important upstream of the regulation of GSDME [39]. The current study identified the binding sites of CHOP in the promoter region of GSDME and verified that GSDME was a transcription target of CHOP. It has been reported that the transcription factor CHOP inhibited BCL-2, mediating the mitochondrial apoptosis pathway. Furthermore, ERS occurs at an early stage and regulates cell death. In the liver, CHOP regulates the transition between multiple forms of cell death transition, including apoptosis to ferroptosis, apoptosis to autophagy, and apoptosis to pyroptosis. This study demonstrated that ERS occurred in the renal IRI, leading to the activation of the transcription factor CHOP (Fig. 6A, B). Moreover, activated CHOP increased the expression levels of GSDME in renal IRI (Fig. 6D). The mRNA expression levels of CHOP and GSDME showed the same trend at different time points of HR (Supplementary Fig. 2D). Subsequently, it was confirmed that CHOP could directly increase the expression levels of GSDME (Fig. 8H). It was further confirmed that CHOP knockout ameliorated apoptosis and GSDME-mediated pyroptosis.

**Fig. 9:**
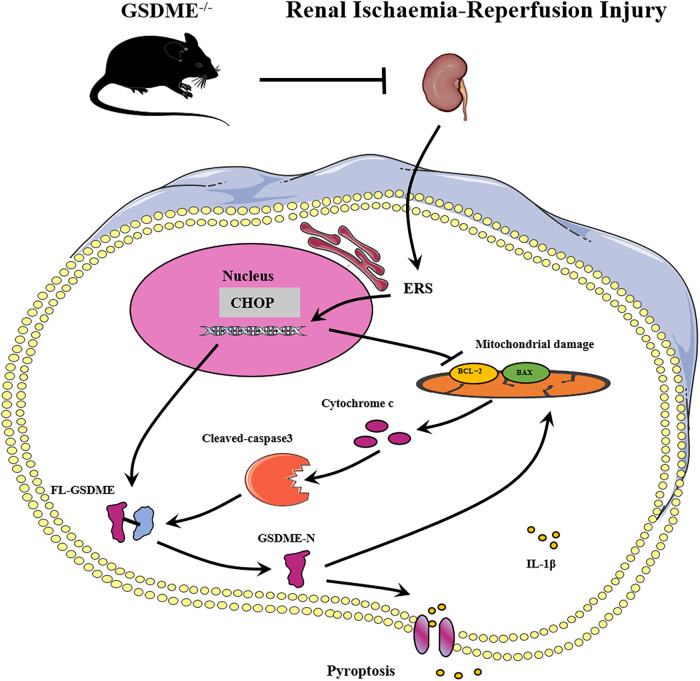
Graphic summary of the mechanisms for GSDME accelerating pyroptosis, mitochondrial damage, and inflammatory response in renal IRI. The transcription factor CHOP activated and up-regulated GSDME, while enhancing Caspase 3 activity through the mitochondrial apoptotic pathway and promoting the renal tubular epithelial cell pyroptosis in the renal ischemia-reperfusion injury. However, GSDME deficiency ameliorated the renal ischemia-reperfusion injury.

Briefly, in IRI, the expression levels of GSDME were elevated, which caused pyroptosis, promoted mitochondrial damage, and released inflammatory factors. However, GSDME deficiency reduced the release of pyroptosis-related proinflammatory cytokines in the renal IRI. It was observed that ERS occurred in the early stage. The transcription factor CHOP inhibited BCL-2, subsequently activating caspase3 in the mitochondrial apoptosis pathway while directly activating GSDME to increase its expression. Ultimately, this provided a mechanistic explanation of the regulator of cell death both apoptosis and pyroptosis, leading to the exacerbation of renal IRI. Moreover, GSDME-N was enriched in the mitochondrial membrane, thereby promoting mitochondrial damage and subsequently activating caspase3, which formed a positive feed-forward damage response loop. Collectively, the important role of the CHOP/Caspase3/GSDME mechanistic axis in regulating apoptosis and pyroptosis in renal IRI was elucidated, as shown in Fig. 9, highlighting its potential as a therapeutic target.

## Material and methods

### Reagents and chemicals

All reagents and antibodies involved in this study were obtained from commercial sources and are listed in Supplementary Table 1.

### Human specimens

Human normal kidney specimens were obtained from paracancerous tissue of a patient with renal cell carcinoma due to radical renal cancer surgery in the Second Affiliated Hospital of Anhui Medical University. The clinical profile of controls are presented in Supplementary Table 2. The related studies were authorized by the Biomedical Ethics Committee of Anhui Medical University (Ethical Approval No. LLSC20190660) and were implemented with the subjects’ written consent and understanding.

### Experimental animals and renal IRI model

The Experimental Animal Center of Anhui Medical University (Anhui, China) furnished adult male C57 mice (20–25 g). Professor Feng Shao of the National Institute of Biological Sciences donated GSDME-deficient (GSDME^−/−^, knockout) C57BL/6J mice. The Gsdme knockout mice were generated by co-microinjection of in vitro-translated Cas9 mRNA and gRNA into the C57BL/6 zygotes (Supplementary Table 6). The methods for this investigation were carried out in accordance with customary standards for animal care, and the project was approved by our university’s committee for experimental animals. The Guidelines for the Care and Use of Laboratory Animals were followed in all procedures. Briefly, Pentobarbital sodium (50 mg/kg, i.p.) was used to completely anesthetize the mice before they were placed on a homeothermic table to maintain their core body temperature at 37 °C. The dorsal costochondral angle was cut in all animals after which the bilateral renal hilums were identified. Then, a non-traumatic vascular clamp was used to ischemia the bilateral kidneys for 40 min, followed by 6 and 24 h of reperfusion. Every mouse was randomly assigned to different treatment groups (*n* = 8 each). Only the bilateral kidneys were visible in the Sham group.

### Cell culture and cell hypoxia/reoxygenation (HR) model

The American Type Culture Collection (ATCC, USA) sold the human renal proximal tubular epithelial cell line (HK-2) that was used in this study. The cell line was cultured in 5% FBS-containing HyCloneTM DMEM/F12 medium, 100 U/ml penicillin, 100 g/ml streptomycin, and 10% fetal bovine serum under 5% CO_2_ and 95% air atmosphere at 37 °C. In line with the procedures previously mentioned [1], a cell H/R model was created. In order to cause hypoxic damage, HK-2 cells were grown for 12 h under hypoxic conditions (1% O_2_, 94% N_2_, and 5% CO_2_) in media devoid of nutrients (glucose-free, serum-free). The plates were then transferred to a normoxic cell incubator (5% CO_2_ and 95% air) for 1, 3 and 6 h after the medium had been refilled once more. In a typical incubator (5% CO_2_ and 95% air), control cells were incubated in full culture media.

### Renal function and histology

Serum was obtained by placing blood into heparinized tubes and centrifuging at 3000 × *g* for 25 min. A serum biochemical autoanalyzer (Hitachi7600 modular chemistry analyzer, Hitachi Ltd, USA) was used in the central laboratory of the Second Affiliated Hospital of Anhui Medical University to measure the Cr and BUN concentrations of the experimental mice. 4% paraformaldehyde was used to fix kidney tissues for 24 h. Following this, the tissues were dehydrated using a graduated ethanol series, cleaned in xylene, and then embedded in paraffin.

### Renal histology and immunohistochemistry

In a standard process, 4 μm-thick paraffin-embedded mouse kidney slices were created. Each animal’s six randomly selected, non-overlapping fields were assessed. The manufacturer’s instructions were followed for doing the immunohistochemical staining. The secondary antibodies were incubated for 30 min at 37 °C before the IL-1β, L18 (Abcam, Cambridge, UK, 1:200) and F4/80+ (CST, Danvers, MA, 1:200) specific antibodies were added. A microscope (Leica, Bensheim, Germany) was used to view the sections after DAB staining and counterstaining with hematoxylin. PAS staining was performed in accordance with industry standards. The percentage of renal tubules that had cell lysis, the loss of the brush border, and the production of casts were used to classify the degree of tissue damage (0, no damage; 1, 25%; 2, 26–50%; 3, 51–75%; 4, >75%).

### Immunofluorescence

Paraffin-embedded mouse kidney sections were prepared by a routine procedure (4 μm). Six randomly chosen, non-overlapping fields per animal were evaluated. The manufacturer’s instructions were followed when performing the immunofluorescence staining. The secondary antibodies were incubated for 30 min at 37 °C after the primary antibodies had been incubated overnight at 4 °C for KIM1, GSDME, GSDMD, BCL-2, BAX, GRP78, CHOP, and Cleaved-caspase3 (1:200). The sections were examined under a microscope (Leica, Bensheim, Germany) after being stained with DAPI. Cells were cultured in six-chamber glass slides, fixed in acetone, and then incubated with antibodies that recognized KIM1, GSDME, GSDMD, BCL-2, BAX, GRP78, CHOP, Cleaved-caspase3, and Cyt-c (1:200) for an entire night. Furthermore, Mitochondrial fluorescent labeling using kits (Beyotime, Jiangsu, China). Cells were washed with PBS and incubated both with goat anti-rabbit IgG-rhodamine and goat anti-mice IgG-rhodamine (Bioss Biotechnology, Beijing, China) for 2 h at room temperature. The cells were counterstained with DAPI and visualized using fluorescence microscopy (Leica, Bensheim, Germany).

### TUNEL assay

Renal cell apoptosis was examined by TUNEL assay using the One step TUNEL Apoptosis Assay Kit from Beyotime Biotechnology (Beyotime, Jiangsu, China). Briefly, cells were fixed with 4% paraformaldehyde in PBS and then exposed to the TUNEL reaction mixture containing TM green–labeled dUTP. Finally, samples were counterstained with 4′,6-diamidino-2-phenylindole (DAPI). TUNEL-positive nuclei were identified by fluorescence microscopy (Leica, Bensheim, Germany).

### Western blot

The kidney sections were ground using a homogenizer or the cells were scratched and lysed in RIPA lysis buffer (Beyotime, Shanghai, China) with proteinase inhibitor (Roche, Switzerland) for 30 min on ice. Protein concentration was quantified using the Bicinchoninic acid assay (Beyotime, Shanghai, China). Protein samples (50 μg) were used to perform immunoblotting as described previously. The specific primary antibodies used were: anti-GSDME (ab215191, Abcam, 1:1000), Anti-cleaved N-terminalDNA5/GSDME (ab222407, Abcam, mouse, 1:1000), Anti-cleavedN-terminalDNA5/GSDME (ab222408, Abcam, human, 1:1000), anti-GSDMD (ab209845, Abcam, 1:1000), anti-caspase-3 (ab184787, Abcam, 1:1000), anti-cleaved-caspase3 (Cat#AF7022, Affinity Biosciences, 1:1000), anti-NLRP3 (ab263899, Abcam, 1:1000), anti-caspase1 (ab207802, Abcam, 1:1000), anti-caspase11 (ab246496, Abcam, 1:1000), anti-caspase8 (ab108333, Abcam, 1:1000), KIM-1 (ab302932, Abcam, 1:1000), anti-Bax (ab32503, Abcam, 1:1000), anti-BCL-2 (ab182858, Abcam, 1:1000), anti-β-actin (ab8226, Abcam, 1:1000), GRP78 (3177, CST, 1:1000), CHOP (2895, CST, 1:1000), phospho-NF-κB P65 (3033, CST, 1:1000), NF-κB p65 (8242, CST, 1:1000), IRDyeTM800-conjugated secondary antibody (Rockland Immunochemicals). Signals were detected using LiCor/Odyssey infrared image system (LI-COR Biosciences, Lincoln, NE, USA). The results were quantitatively analyzed using Image J software (National Institutes of Health).

### Renal RNA extraction and real-time PCR

Total RNA of kidney tissues and cells was isolated using TRIzol Reagent (Invitrogen, Carlsbad, CA). Complementary DNA (cDNA) was synthesized using the PrimeScript RT Reagent Kit (TaKaRa, Tokyo, Japan). Real-time PCR was performed using BioRad iQ SYBR Green supermix with Opticon2 (Bio-Rad, Hercules, CA). The sequences of the PCR primers in this study are listed in Supplementary Table 3.

### Mitochondrial membrane voltage potential and ROS

Mitochondrial membrane potential (MMP) was evaluated by JC-1 (Beyotime, Jiangsu, China) staining. ROS detection by C11 lipid peroxidation and DCFH (Beyotime, Jiangsu, China). The treated HK-2 cells were stained according to the JC-1 kit instructions and then incubated at 37 °C for 30 min before detection by confocal microscopy. In healthy mitochondria, JC-1 aggregates in the mitochondrial matrix emitted strong red fluorescence. In contrast, in unhealthy mitochondria, JC-1 appeared in the cytoplasm, generating green fluorescence owing to a decrease in mitochondrial membrane potential. Additionally, we performed DCFH staining on frozen sections of renal tissue to evaluate the level of intracellular ROS. Immunofluorescence images were obtained using a confocal laser scanning microscope (Leica, Bensheim, Germany). All results were analyzed using ImageJ software, and the average fluorescence density values of the control group were set as the reference.

### Cell Plasma Membrane Staining Kit with DiI (red fluorescence)

Adherent cells were seeded on cell crawls. Aspirate the cell culture solution and wash the cells 2 times with PBS. Add the appropriate volume of cell membrane staining working solution and shake gently so that the dye evenly covers all cells. Incubate the cells at 37 °C away from light for 5–20 min, the optimal incubation time varies for different cells. Remove the working solution for cell membrane staining, wash with PBS for 2–3 times, and then add 37 °C pre-warmed cell culture solution to be observed under fluorescence microscope.

### LDH assay

Cells were cultured in 96-well plates and then subjected to different treatments. One hour before assay, 20 μl of LDH release agent was added to the culture medium. LDH release levels were assessed by using LDH Cytotoxicity Assay Kit II (Beyotime, Shanghai, China), according to the manufacturer instructions.

### Calcein/PI staining

The Calcein/PI staining kit (Beyotime, Shanghai, China) was used to assess the pyroptotic cell death of cells. After coincubation with Calcein/PI staining kit (1 μl/ml) for 30 min at 37 °C, followed by resuspension in PBS. After that, the cells visualized using fluorescence microscopy (Leica, Bensheim, Germany).

### Cell viability assay

After hypoxia/reoxygenation in 96-well culture plates, HK-2 cells were incubated in Dulbecco’s modified Eagle’s medium-F12 medium supplemented with 10% fetal bovine serum. For 2 h at 37 °C, cells were treated with 10 ml of CCK-8 (Beyotime, Shanghai, China). Molecular Devices’ SpectraMax Paradigm Multi-Mode Microplate Reader (Sunnyvale, CA) was used to measure the color reaction at 450 nm.

### Binding site prediction

The position weight matrix technique from JASPAR [40] was used to scan the promoter regions of a subset of mouse Gsdme genes in order to determine the probable CHOP binding sites. The promoter regions were defined as being between 2000 and 500 from the gene’s transcriptional start point.

### siRNA-mediated gene knockdown

HK-2 cells were planted on a plate and cultivated to 80% confluence. Following the manufacturer’s instructions, cells were transfected with siRNA or siRNA serving as a negative control (30 nM/each siRNA) using Lipofectamine 3000 Reagent (catalog number: L3000001, Thermo Fisher Scientific). siCHOP and SiNegative were from (Hanbio, Shanghai, China). The sequences of the PCR primers in this study are listed in Supplementary Table 4.

### Plasmid construction and transfection

HK-2 cells were planted on 6-well plate and cultivated to 50% confluence. Following the manufacturer’s instructions, cells were transfected with GSDME (NM_004403 (1-270aa)) overexpression plasmid (2 ug/each well) using Lipofectamine 3000 Reagent (catalog number: L3000001, Thermo Fisher Scientific). GSDME (NM_004403 (1-270aa)) overexpression plasmid was from (Jikai, Shanghai, China). Information sheet is constructed with the overexpression vector (GSDME-N) (Supplementary Table 5).

### Chromatin immunoprecipitation assay (ChIP)

As instructed by the manufacturer, the SimpleChIP Enzymatic Chromatin IP Kit (catalog number: 9003, CST) was used for the ChIP assay. The normal rabbit IgG (CST, catalog number: 2729, 5 g for a single assay) or the CHOP antibody (CST, catalog number: 2895, 1:100) were used to precipitate the sheared chromatin. The primers (Forward primer sequence5’-3’:AGCTTTCCCTCTTGCACACG Reverse primer sequence5’-3’:GCTGGTGCTCAAACATTGCT) and template for qPCR were made from purified precipitated DNA.

### Statistical analysis

Data are displayed as means ± SEM. An unpaired two-tailed test was used to analyze statistical significance. Between-group comparisons were done using the Student’s *t* test, and multiple comparisons were done using a one-way ANOVA with the Bonferroni post hoc test. To find significant differences between two groups, non-normally distributed data were analyzed using the Mann–Whitney *U* test. *p* < 0.05 was regarded as statistically significant for all two-tailed statistical tests. With the help of Graphpad Prism 9.0, the data were plotted.

### Reporting summary

Further information on research design is available in the Nature Research Reporting Summary linked to this article.

### Supplementary information

Supplementary figures and tables

Original Data File

Reporting Summary

## Data Availability

All datasets generated and analyzed during this study are included in this published article and its Supplementary Information files. Additional data are available from the corresponding author on reasonable request.
