# Supplementary material for: CHOP-mediated Gasdermin E expression promotes pyroptosis, inflammation, and mitochondrial damage in renal ischemia-reperfusion injury
Source: Cell Death Dis. 2024 Feb 22;15(2):163. doi: 10.1038/s41419-024-06525-9 (PMC10883957; doi:10.1038/s41419-024-06525-9)
Supplement: Supplementary file 1 — Supplementary figures and tables [file 41419_2024_6525_MOESM1_ESM.docx]

**CHOP-mediated Gasdermin E expression promotes pyroptosis, inflammation, and mitochondrial damage in renal ischemia-reperfusion injury**

Nannan Ma^1,†^, Hao Lu^2,†^, Ning Li^3,†^, Weijian Ni^4,5,2†^, Wenbo Zhang^1^, Qiang Liu^1^, Wenzheng Wu^1^, Shichao Xia^2^, Jiagen Wen^2,*^, Tao Zhang^1,*^

^1^ Department of Urology, The Second Affiliated Hospital of Anhui Medical University, Hefei, Anhui, People’s Republic of China

^2^ Inflammation and Immune Mediated Diseases Laboratory of Anhui Province, Anhui Institute of Innovative Drugs, School of Pharmacy, Anhui Medical University, Hefei, Anhui, People’s Republic of China

^3^ Department of Nephropathy, The Zhongda Affilicated Hospital of Southeast University, Nanjing, Jiangsu, People’s Republic of China

^4^ Department of Pharmacy, Centre for Leading Medicine and Advanced Technologies of IHM, Anhui Provincial Hospital, The First Affiliated Hospital of USTC, Division of Life Sciences and Medicine, University of Science and Technology of China, Hefei, Anhui, People’s Republic of China

^5^ Anhui Provincial Key Laboratory of Precision Pharmaceutical Preparations and Clinical Pharmacy, Hefei, Anhui, People’s Republic of China

*Corresponding author

Jiagen Wen, School of Pharmacy, Anhui Medical University, Hefei, Anhui,230032, China. E-mail address: jiagenwen@ahmu.edu.cn

Tao Zhang, Department of Urology, The Second Affiliated Hospital of Anhui Medical University, Hefei, Anhui, 230032, China. E-mail address: [zhangtao@ahmu.edu.cn](mailto:zhangtao@ahmu.edu.cn)

^†^ These authors contributed equally to this work.

**Contents:**

**Supplemental Figure 1**

**Supplemental Figure 2**

**Supplementary Table 1 - Reagents used in the study**

**Supplementary Table 2 - Clinical characteristics of the patients**

**Supplementary Table 3 - Specific primers for quantitative RT-PCR**

**Supplementary** **Table 4 - Primers for CHOP knockout（siRNA sequences used for gene knockdown）**

**Supplementary Table 5 - Information sheet was constructed with the overexpression vector（GSDME-N)**

**Supplementary Table 6 - The Gsdme knockout mice were generated by co-microinjection of in vitro-translated Cas9 mRNA and gRNA into the C57BL/6 zygotes.**


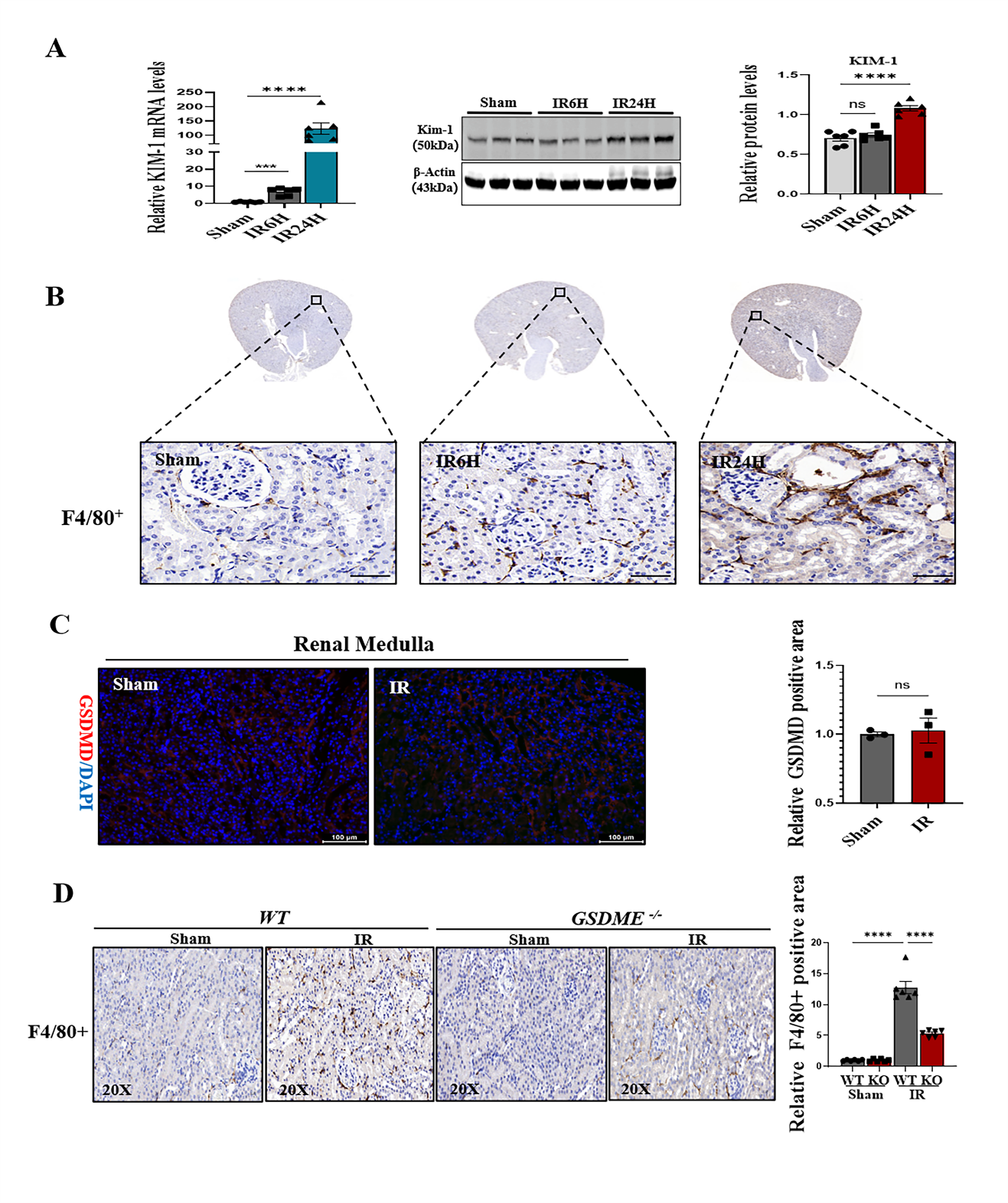


**Supplemental Figure 1**

**A.** KIM1 protein and mRNA were determined by western blotting and qPCR, respectively, in different groups. n = 6/group. **B.** Representative immunohistochemical staining of F4/80+ in IRI mice kidney,compared with Sham. **C.** Representative immunofluorescence image of GSDMD was expressed in mice kidney medulla. n= 6/group Scale bar: 100 μm. **D.** Representative immunohistochemistry (IHC) image of F4/80+ was expressed in different groups, n=6/group, Scale bar:50μm. For all panels, P value was determined by unpaired two-tailed Student’s t test or one-way ANOVA with Bonferroni post hoc test for multiple comparisons. Data are expressed as mean ± SEM. Quantifification on the blots derive from samples of the same experiment and gels/blots were processed in parallel.ns,not signifificant p>0.05 *p<0.05 **p<0.01 ***p<0.001 ****p<0.0001.


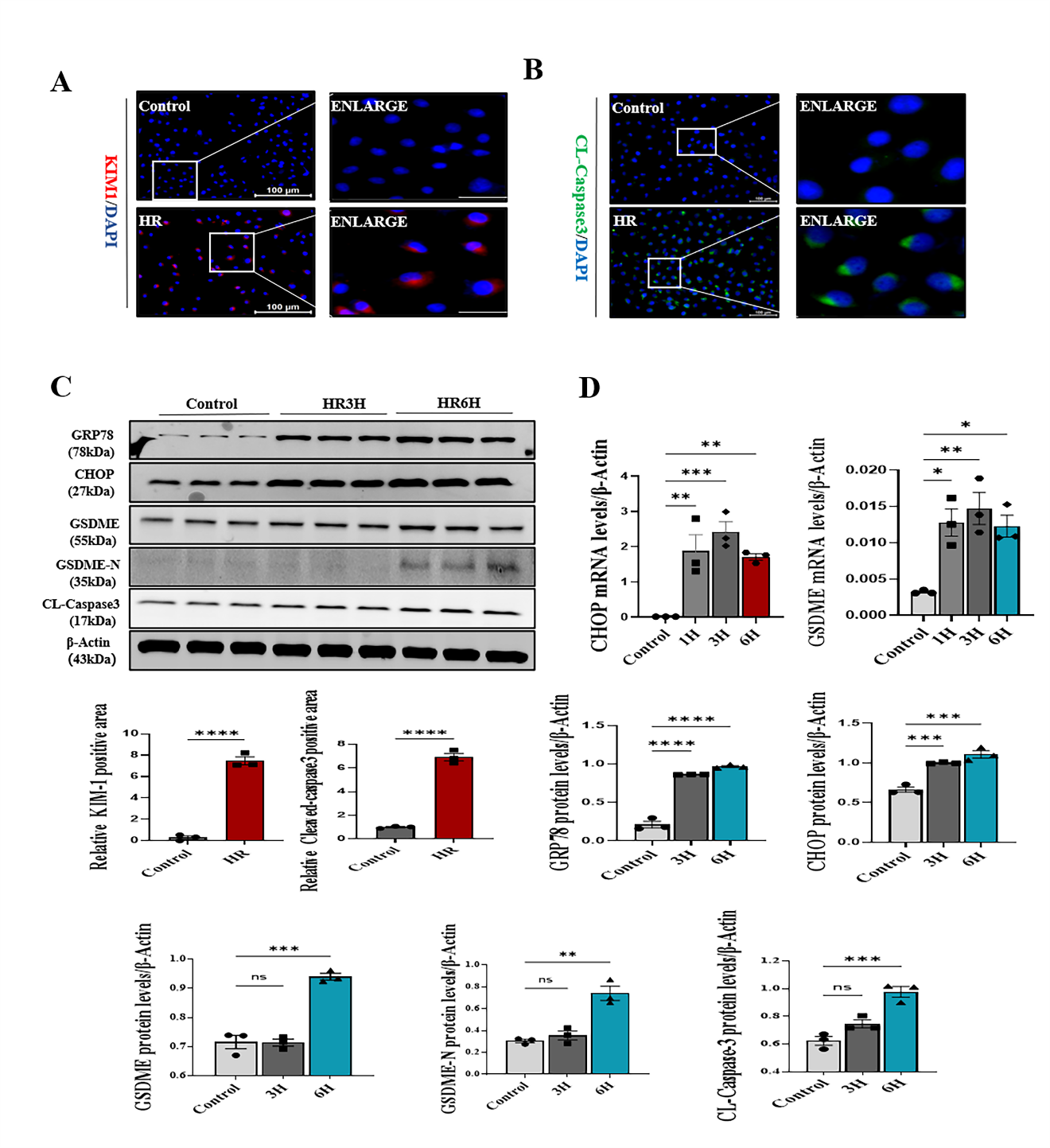


**Supplemental Figure 2**

**A.** Representative immunofluorescence image of KIM-1 in HK2,Red fluorescence represents KIM1,the protein fluorescence intensity/area in each group are shown, n=3/group, Scale bar:100μm;Scale bar:20 μm (magnification). **B.** Representative immunofluorescence image of Cleaved-caspase3 in HK2,Green fluorescence represents Cleaved-caspase3,the protein fluorescence intensity/area in each group are shown, n=3/group, Scale bar:100μm;Scale bar:20 μm (magnification). **C.** Western blotting analysis of GRP78,CHOP,GSDME,GSDME-N and Cl-caspase3 expression. n = 3/group. **D.** Quantitative polymerase chain reaction (qPCR) analysis of the gene expression relative to CHOP as well as GSDME at different time points of HR. n = 3/group. For all panels, P value was determined by unpaired two-tailed Student’s t test or one-way ANOVA with Bonferroni post hoc test for multiple comparisons. Data are expressed as mean ± SEM. Quantification on the blots derive from samples of the same experiment and gels/blots were processed in parallel. ns, not significant p>0.05 *p<0.05 **p<0.01 ***p<0.001 ****p<0.0001.

**Supplementary Table 1.**

The specifific primary antibodies used were: anti-GSDME (ab215191, Abcam, 1:1000), Anti-N-terminal DNA5/GSDME (ab222407, Abcam, mouse, 1:1000), Anti-cleaved N-terminal DNA5/GSDME (ab222408, Abcam, human, 1:1000), anti-GSDMD (ab209845, Abcam, 1:1000), anti-caspase-3 (ab184787, Abcam, 1:1000), anti-cleavedcaspase-3 (Cat#AF7022, AffinityBiosciences, 1:1000), anti-NLRP3 (ab263899, Abcam, 1:1000), anti-caspase1 (ab207802, Abcam, 1:1000), anti-caspase11 (ab246496, Abcam, 1:1000), anti-caspase8 (ab108333, Abcam, 1:1000), KIM-1 (ab302932, Abcam, 1:1000), anti-Bax (ab32503, Abcam, 1:1000), anti-BCL-2 (ab182858, Abcam, 1:1000) and anti-β-actin (ab8226, Abcam, 1:1000), GRP78 (3177, CST, 1:1000), CHOP (2895, CST, 1:1000), phospho-NF-κB p65 (3033, CST, 1:1000), NF-κB p65 (8242, CST, 1:1000); Cytochrome C (Proteintech, Cat No. 66264-1-Ig); IL-1β, L-18 (Abcam, Cambridge, UK, 1:200); F4/80+ (CST, Danvers, MA, 1:200); Mitochondrial fluorescent labelling using kits (Beyotime, Jiangsu, China); Goat anti-rabbit IgG-rhodamine and goat anti-mice IgG-rhodamine (Bioss Biotechnology, Beijing, China); Periodic acid Schiff (PAS) were obtained from Nanjing Jiancheng Bioengineering Institute (Nanjing, China); TUNEL Apoptosis Assay Kit from Beyotime Biotechnology (Beyotime, Jiangsu, China); RIPA lysis buffer (Beyotime, Shanghai, China) with proteinase inhibitor (Roche, Switzerland) ; TRIzol Reagent (Invitrogen, Carlsbad, CA); SYBR Green supermix with Opticon2 (Bio-Rad, Hercules, CA); JC-1 (Beyotime, Jiangsu, China); C11 lipid peroxidation and DCFH (Beyotime, Jiangsu, China); LDH Cytotoxicity Assay Kit II (Beyotime, Shanghai, China); Calcein/PI staining kit (Beyotime, Shanghai, China); CCK-8 (Beyotime, Shanghai, China); Lipofectamine 3000 Reagent (catalog number: L3000001, Thermo Fisher Scientific); siCHOP and SiNegative were from (Hanbio, Shanghai, China); SimpleChIP Enzymatic Chromatin IP Kit（catalog number: #9003）was purchased from Cell Signaling Technology (CST, Danvers, MA).

|  | **Patient1** | **Patient2** | **Patient3** | **Patient4** | **Patient5** |
| --- | --- | --- | --- | --- | --- |
| **Sex** | F | F | F | F | F |
| **Age** | 48 | 46 | 61 | 57 | 61 |
| **BMI** | 27.43 | 24.09 | 25.32 | 28.52 | 25.69 |
| **Hypertension** | no | no | no | no | no |
| **Diabetes** | no | no | no | no | no |
| **CRE** | 41 | 44 | 43 | 54 | 89 |
| **BUN** | 4.03 | 6.68 | 5.13 | 5.07 | 6.54 |
| **Surgery** | R-RN | R-RN | R-RN | L-RN | R-RN |

**Supplementary Table 2. Clinical characteristics of the patients (Normal)**

|  | **Patient6** | **Patient7** | **Patient8** | **Patient9** | **Patient10** |
| --- | --- | --- | --- | --- | --- |
| **Sex** | F | F | M | M | M |
| **Age** | 52 | 40 | 55 | 48 | 46 |
| **BMI** | 19.92 | 23.05 | 20.55 | 21.13 | 29.96 |
| **Hypertension** | no | no | no | no | no |
| **Diabetes** | no | no | no | no | no |
| **CRE** | 46 | 56 | 84 | 95 | 62 |
| **BUN** | 3.96 | 3.64 | 6.27 | 6.28 | 3.66 |
| **Surgery** | R-RN | R-RN | L-RN | R-RN | R-RN |

|  | **Patient11** | **Patient12** | **Patient13** | **Patient14** |  |
| --- | --- | --- | --- | --- | --- |
| **Sex** | M | M | M | M |  |
| **Age** | 47 | 57 | 36 | 54 |  |
| **BMI** | 19.14 | 19.59 | 25.95 | 23.12 |  |
| **Hypertension** | no | no | no | no |  |
| **Diabetes** | no | no | no | no |  |
| **CRE** | 76 | 79 | 82 | 55 |  |
| **BUN** | 6.49 | 5.41 | 5.93 | 4.36 |  |
| **Surgery** | L-RN | R-RN | L-RN | R-RN |  |

Human kidney samples were obtained from 7 male and 7 female patients undergoing Radical surgery for renal cancer (RN) procedure in the Second Affiliated Hospital of Anhui Medical University; F: Female; M: Male; CRE: creatinine; BUN: urea nitrogen; BMI: body mass index; L: left; R: right.

**Supplementary Table 3. Specific primers for quantitative RT-PCR**

| **Mouse Genes** | **Forward primer (5′–3′)** | **Reverse primer (5′–3′)** |
| --- | --- | --- |
| β-actin  KIM-1  MCP-1  TNF-α  IL-1β  IL-6  IL-18  GRP78  CHOP | CATTGCTGACAGGATGCAGAA  CAGGGAAGCCGCAGAAAA  CTTCTGGGCCTGCTGTTCA  CATCTTCTCAAAATTCGAGTGACAA  GCTTCAGGCAGGCAGTAT  GAGGATACCACTCCCAACAGACC  GACTCTTGCGTCAACTTCAAGG  ACTTGGGGACCACCTATTCCT  AAGCCTGGTATGAGGATCTGC | ATGGTGCTAGGAGCCAGAGC  GAGACACGGAAGGCAACCAC  CCAGCCTACTCATTGGGATCA  TGGGAGTAGACAAGGTACAACCC  ACAAACCGCTTTTCCATCT  AAGTGCATCATCGTTGTTCATACA  CAGGCTGTCTTTTGTCAACGA  GTTGCCCTGATCGTTGGCTA  TTCCTGGGGATGAGATATAGGTG |

| **Human Genes** | **Forward primer (5′–3′)** | **Reverse primer (5′–3′)** |
| --- | --- | --- |
| GSDMD  GSDME  GRP78  CHOP  TNF-α  IL-6  IL-1β  MCP-1  β-actin | GTGTGTCAACCTGTCTATCAAGG  ACATGCAGGTCGAGGAGAAGT  CATCACGCCGTCCTATGTCG  GGAAACAGAGTGGTCATTCCC  CCCAGGGACCTCTCTCTAATCA  CGGGAACGAAAGAGAAGCTCTA  ACTACAGCAAGGGCTTCAGG  AGCAGCAAGTGTCCCAAAGA  CGCCGCCAGCTCACCATG | CATGGCATCGTAGAAGTGGAAG  TCAATGACACCGTAGGCAATG  CGTCAAAGACCGTGTTCTCG  CTGCTTGAGCCGTTCATTCTC  GCTACAGGCTTGTCACTCGG  GAGCAG CCCCAGGGAGAA  CATATCCTGTCCCTGGAGGT  GGTGGTCCATGGAATCCTGA  CACGATGGAGGGGAAGACGG |

**Supplementary Table 4. Specific primers for quantitative RT-PCR**

**（siRNA sequences used for gene knockdown）**

| **Human Genes** | **Forward primer (5′–3′)** | **Reverse primer (5′–3′)** |
| --- | --- | --- |
| NC  si-CHOP-1  si-CHOP-2  Si-CHOP-3 | UUCUCCGAACGUGUCACGUTT  GUCCUGUCUUCAGAUGAAATT  GGAGAACCAGGAAACGGAATT  GCUAGCUGAAGAGAAUGAATT | ACGUGACACGUUCGGAGAATT  UUUCAUCUGAAGACAGGACTT  UUCCGUUUCCUGGUUCUCCTT  UUCAUUCUCUUCAGCUAGCTT |

**Supplementary Table 5. Information sheet was constructed with the overexpression vector**

**Overexpression vector constructs gene information**

Gene Name: GSDME (NM _ 004403 (1-270 aa))

species: Human

Carrier information:

Name of the vector: GV 657

Component order: CMV enhancer-MCS-3flag-polyA-EF1A-zsGreen-sv 40-puromycin

Clone site: BamHI / KpnI

Control number: CON468

Acquisition of the target gene fragments primer ID seq

| **ID** | **seq** |
| --- | --- |
| GSDME(99784-1)-p1 | CACACTGGACTAGTGGATCCCGCCACCATGTTTGCCAAAGCAACCAG |
| GSDME(99784-1)-p2 | AGTCACTTAAGCTTGGTACCGAATCTGGCATGTCTATG |

Primer description: containing the exchange pairing base, restriction site, and containing part of the 5 ′ end of the target gene for catching the target gene by PCR.

PCR product size: 859

**Recombinant plasmid constructs**

Products were exchanged into the linearized expression vector

Primers were identified by PCR ID seq

| **ID** | **seq** |
| --- | --- |
| GSDME(99784-1)-p1 | CACACTGGACTAGTGGATCCCGCCACCATGTTTGCCAAAGCAACCAG |
| GSDME(99784-1)-p3 | GAGCGTATGTTAGTACTATCG |

Identification of the recombinant clones by PCR

PCR product size of positive transformants: 1023

Results of sequencing and result analysis of positive clones:

Comparison results:

AGCGGCCGCCACTGTGCTGGATATCTGCAGAATTCCACCACACTGGACTAGTGGATCCCGCCACCATGTTTGCCAAAGCAACCAGGAATTTTCTTAGAGAAGTTGATGCTGATGGTGACCTGATTGCAGTATCAAATCTGAATGACTCTGATAAGTTACAGCTTCTAAGTCTGGTGACAAAAAAGAAGAGATTCTGGTGCTGGCAGAGACCCAAGTACCAGTTTTTATCCCTCACCCTTGGCGATGTACTCATAGAAGACCAATTTCCGAGTCCAGTGGTCGTGGAGTCGGACTTTGTGAAATACGAGGGCAAGTTTGCAAACCACGTGAGTGGAACCCTGGAGACTGCACTGGGGAAGGTCAAGCTGAACCTGGGGGGCAGCAGCCGCGTAGAGAGCCAGTCTTCATTTGGAACCCTGAGGAAGCAGGAGGTGGATTTGCAGCAGCTCATCAGAGACTCTGCCGAGAGAACAATAAATCTGAGAAACCCTGTGCTCCAGCAGGTGCTGGAAGGAAGGAATGAGGTCCTGTGCGTTTTGACACAGAAGATCACGACGATGCAGAAGTGTGTGATCTCTGAGCACATGCAGGTCGAGGAGAAGTGTGGTGGCATCGTGGGCATCCAGACCAAGACGGTGCAGGTGTCAGCGACGGAGGATGGGAATGTCACCAAGGACTCCAACGTGGTGCTGGAGATCCCAGCTGCCACCACCATTGCCTACGGTGTCATTGAGTTATACGTGAAACTGGACGGCCAGTTCGAGTTCTGCCTTCTCCGAGGGAAGCAAGGTGGCTTCGAGAACAAGAAGAGAATTGACTCTGTCTACCTGGACCCCCTGGTCTTTCGAGAGTTTGCATTCATAGACATGCCAGATTCGGTACCAAGCTTAAGTGACTACAAGGATGACGATGACAAGGATTACAAAGACGACGATGATAAGGACTATAAGGATGATGACGACAAATCTAGATAGTTAATTAAACCGGTAATAAAATATCTTTATTT.

**Supplementary Table 6**

**Gene：*Gsdme***

**Background: C57BL/6**

**The *Gsdme* knockout mice were generated by co-microinjection of in vitro-translated Cas9 mRNA and gRNA into the C57BL/6 zygotes.**

Primer：

CCATTACTGTGGCTAAAGAGGGG mouse Gsdme KO-F

TCCTAAACTCCTGCGGAAGACA mouse Gsdme KO-R

GCCTAGCTTTGAAGTCTAATGTTGTCCAG mouse Gsdme KO-R2


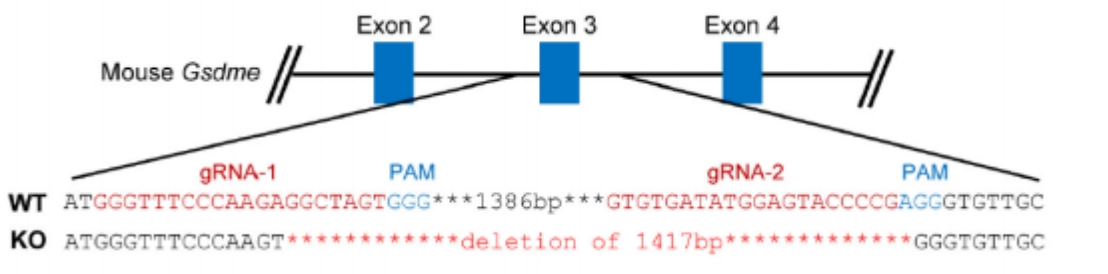


|  | WT mice | Gsdme KO mice |
| --- | --- | --- |
| Gsdme KO-F + Gsdme KO-R | NO | Yes 202bp |
| Gsdme KO-F + Gsdme KO-R2 | Yes 679bp | NO |

PCR procedure：

Gsdme KO-F + Gsdme KO-R

Tag mix

95℃ 5min

95℃ 30s

60℃ 30s 33 cycles

72℃ 30s

72℃ 10min

Gsdme KO-F + Gsdme KO-R2

95℃ 5min

95℃ 30s

60℃ 30s 33 cycles

72℃ 50s

72℃ 10min
