# Supplementary material for: CHOP-mediated Gasdermin E expression promotes pyroptosis, inflammation, and mitochondrial damage in renal ischemia-reperfusion injury
Source: Cell Death Dis. 2024 Feb 22;15(2):163. doi: 10.1038/s41419-024-06525-9 (PMC10883957; doi:10.1038/s41419-024-06525-9)

Full and uncropped western

Figure 1H

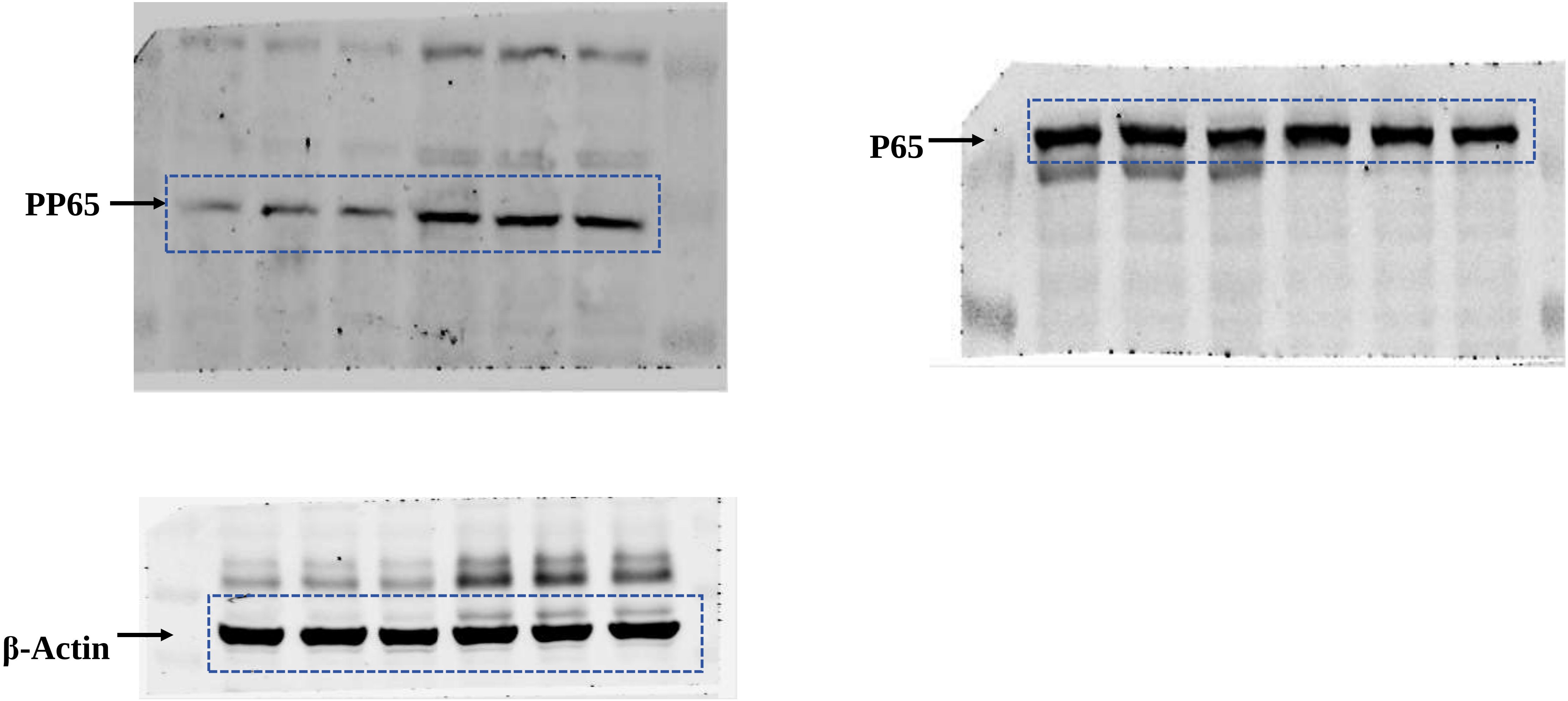

Full and uncropped western

Figure 2B

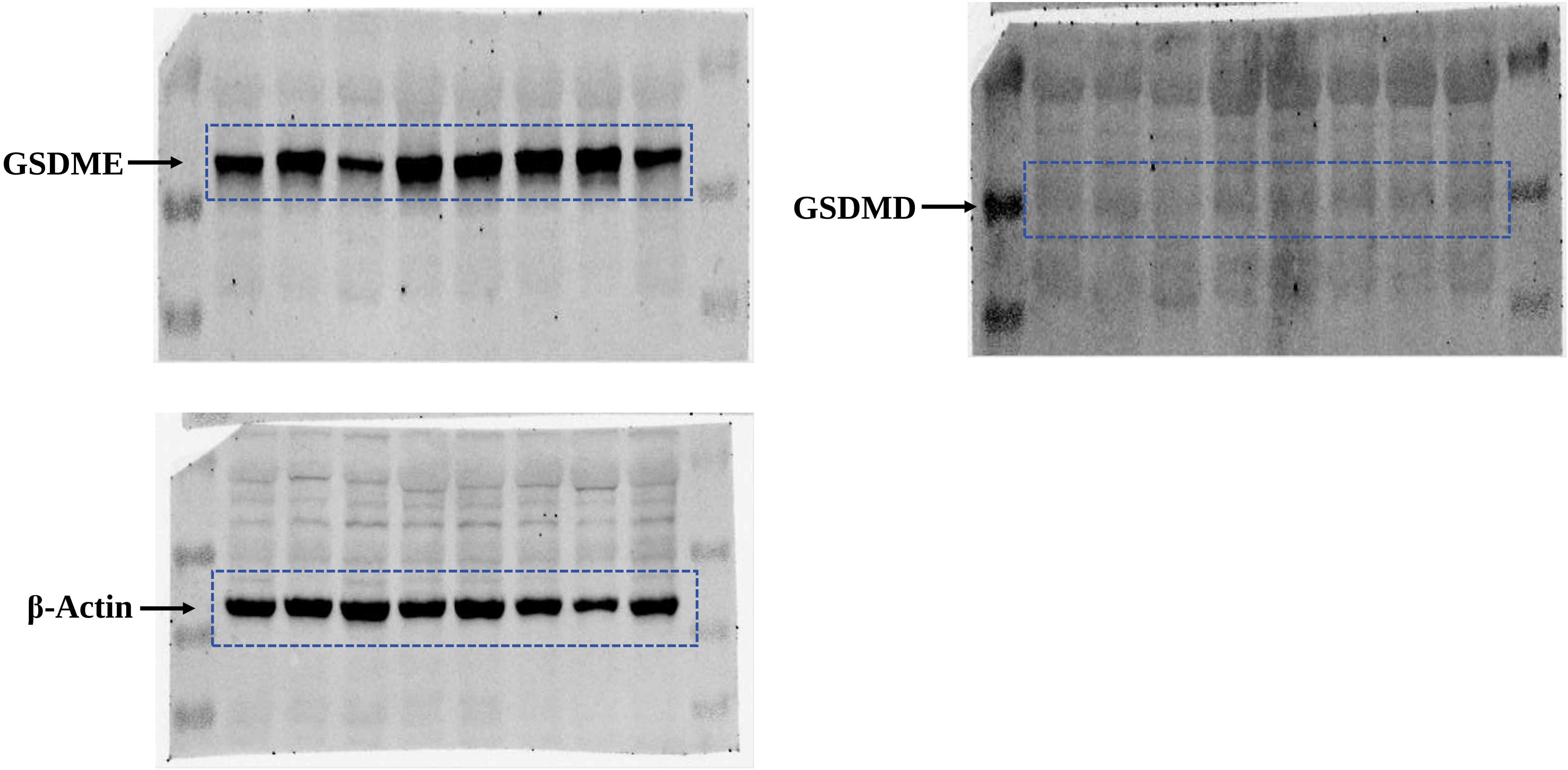

Full and uncropped western

Figure 2G

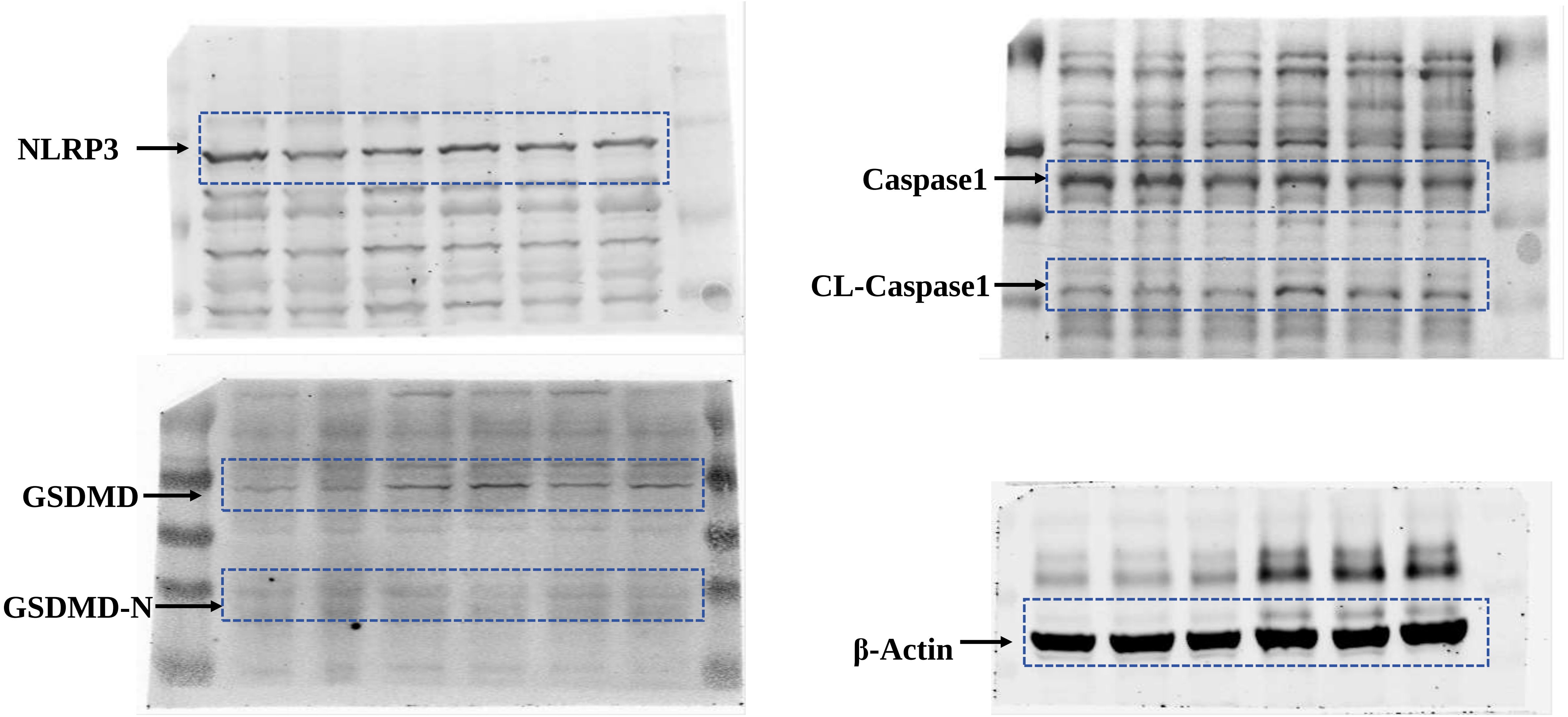

Full and uncropped western

Figure 2G

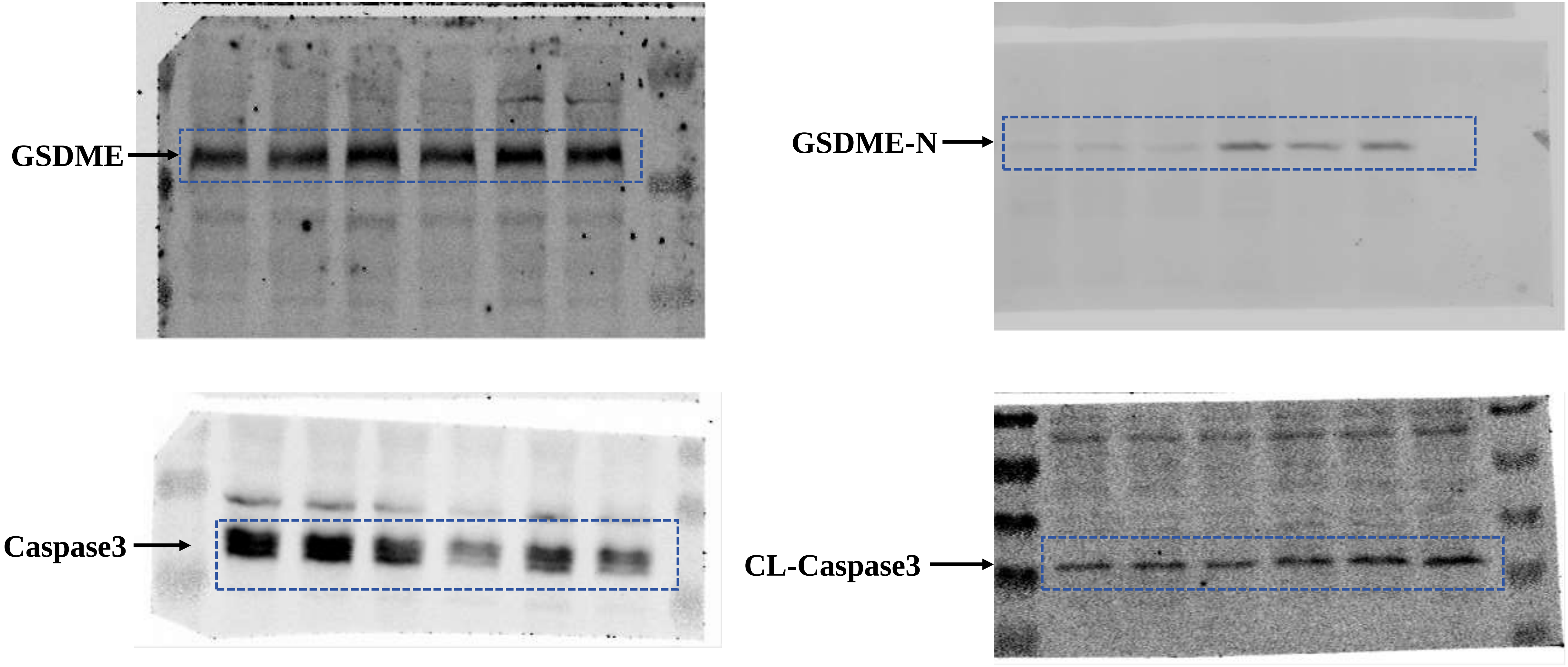

Full and uncropped western

Figure 2G

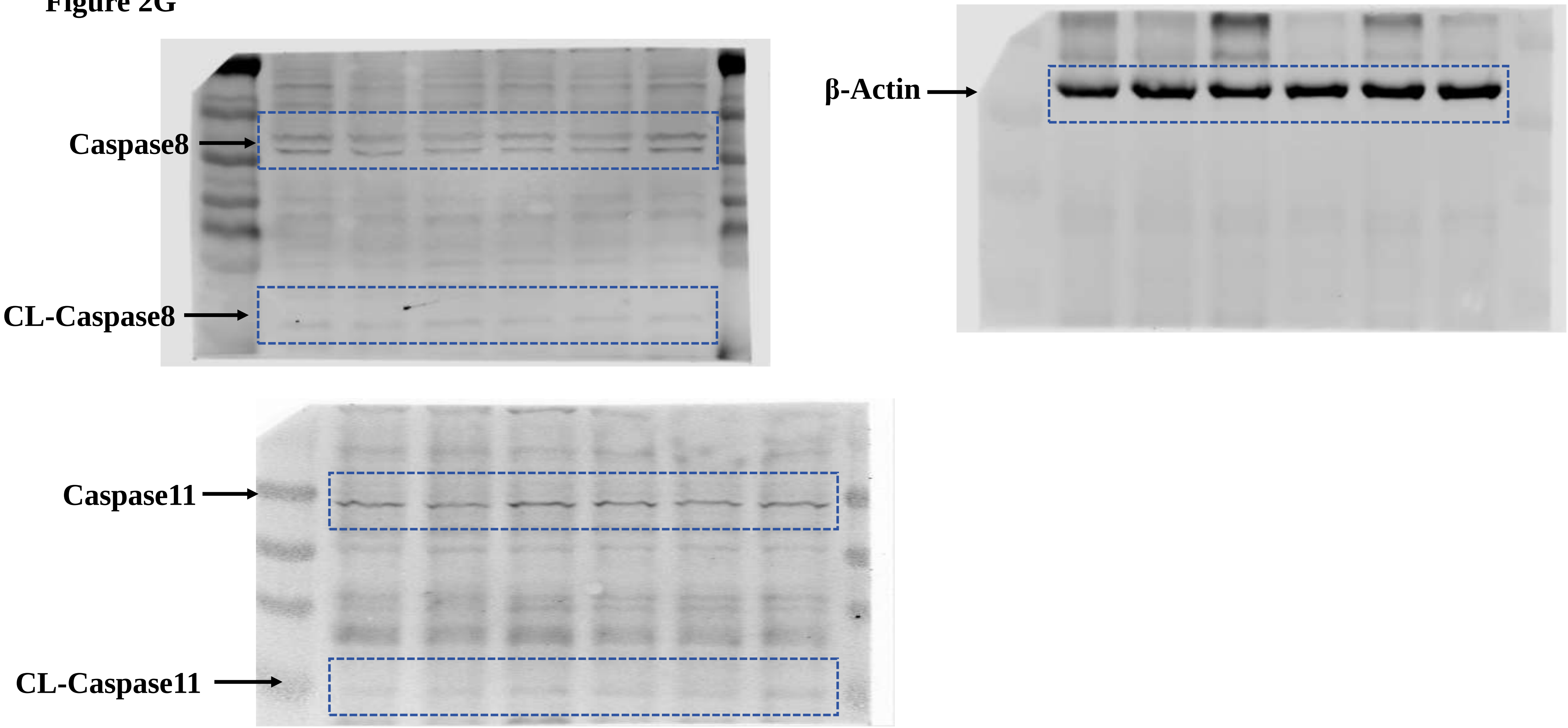

Full and uncropped western

Figure 3I

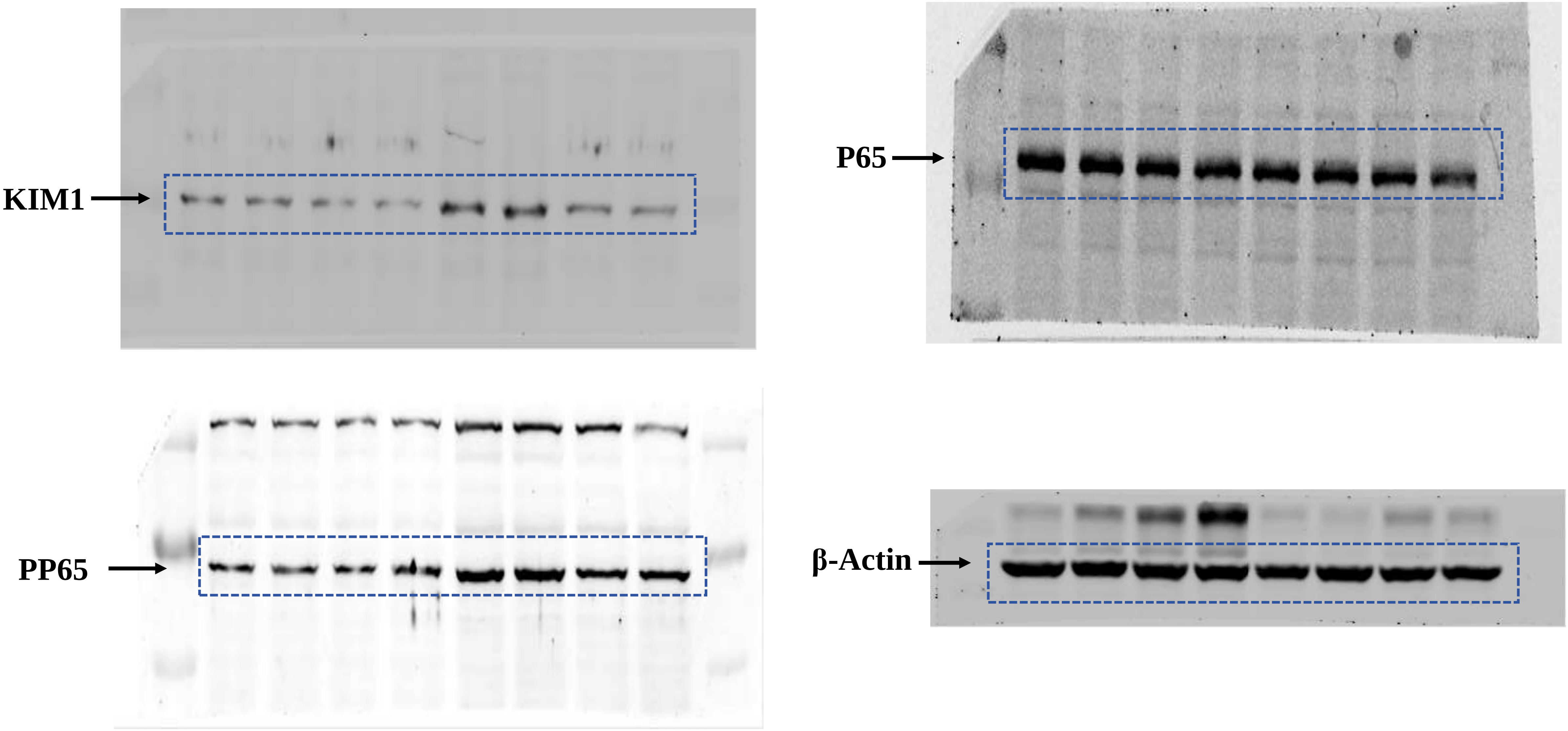

Full and uncropped western

Figure 3J

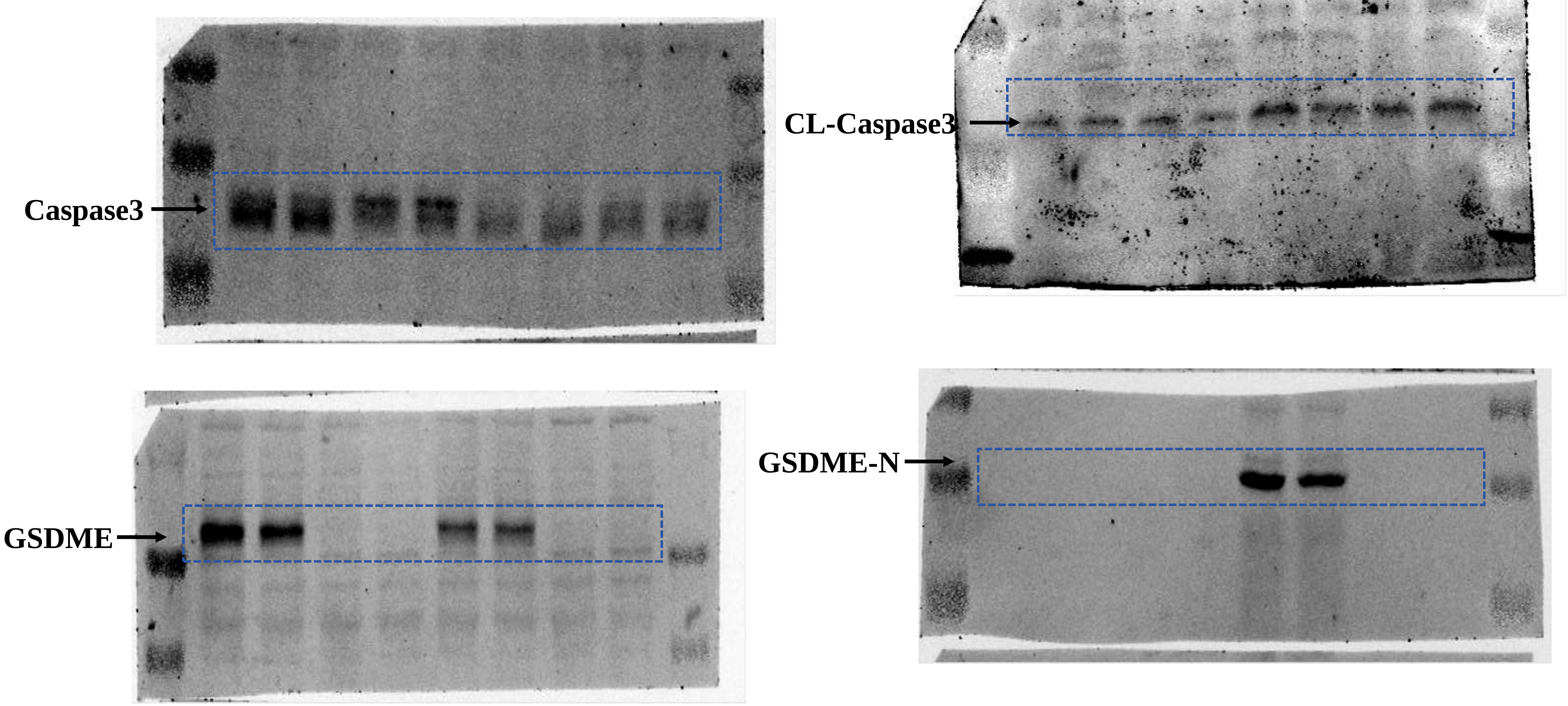

Full and uncropped western

Figure 3J

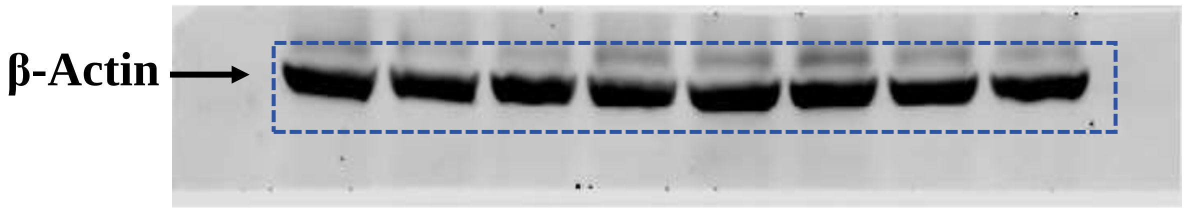

Full and uncropped western

Figure 4B

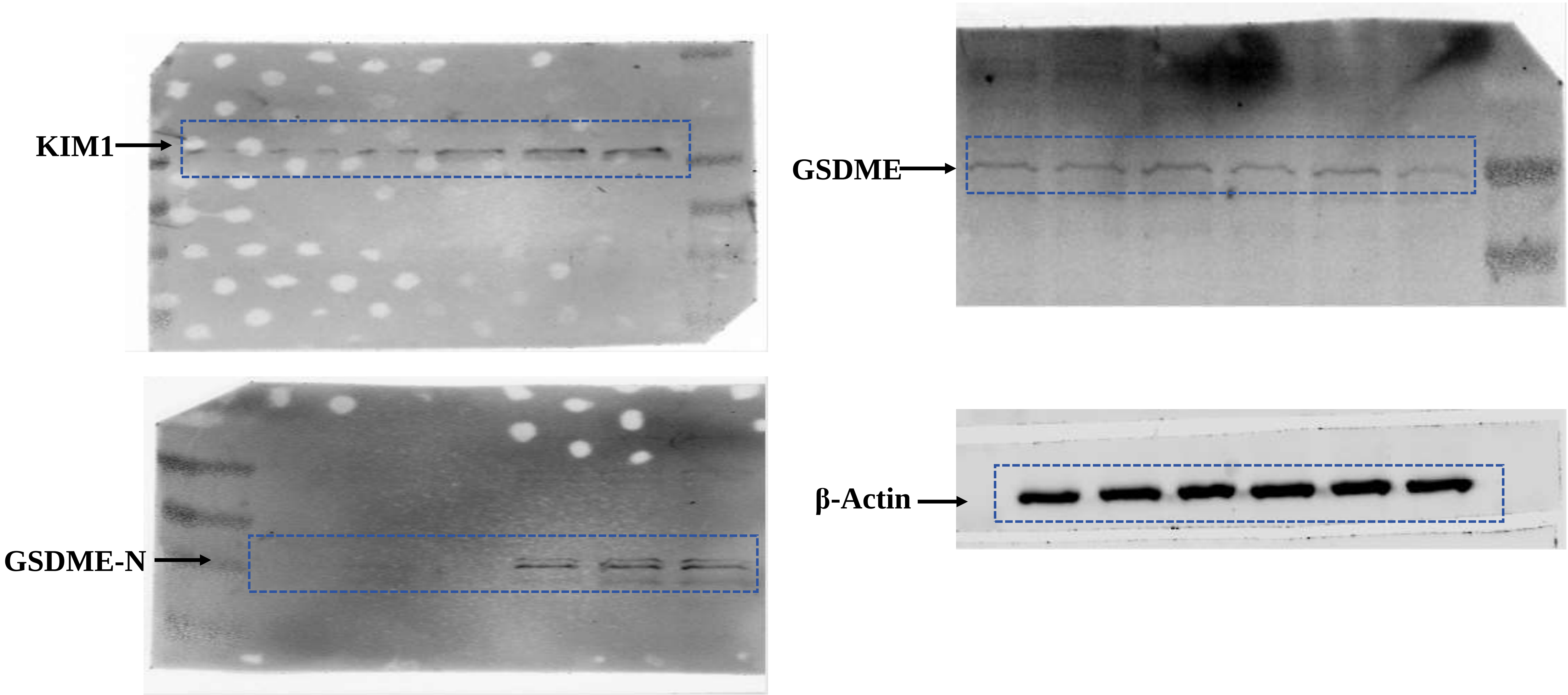

Full and uncropped western

Figure 5D

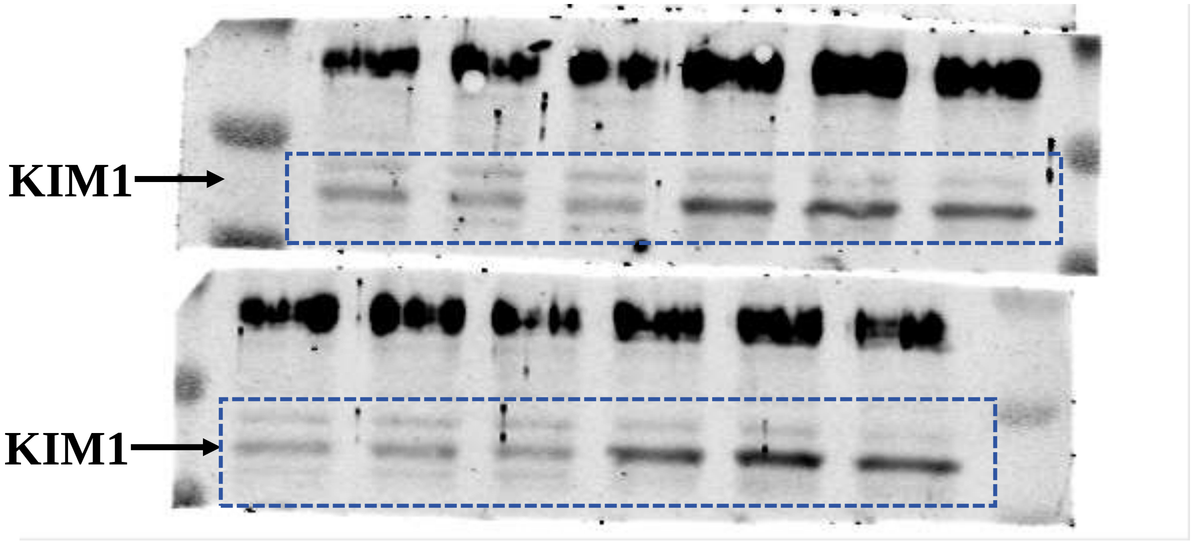

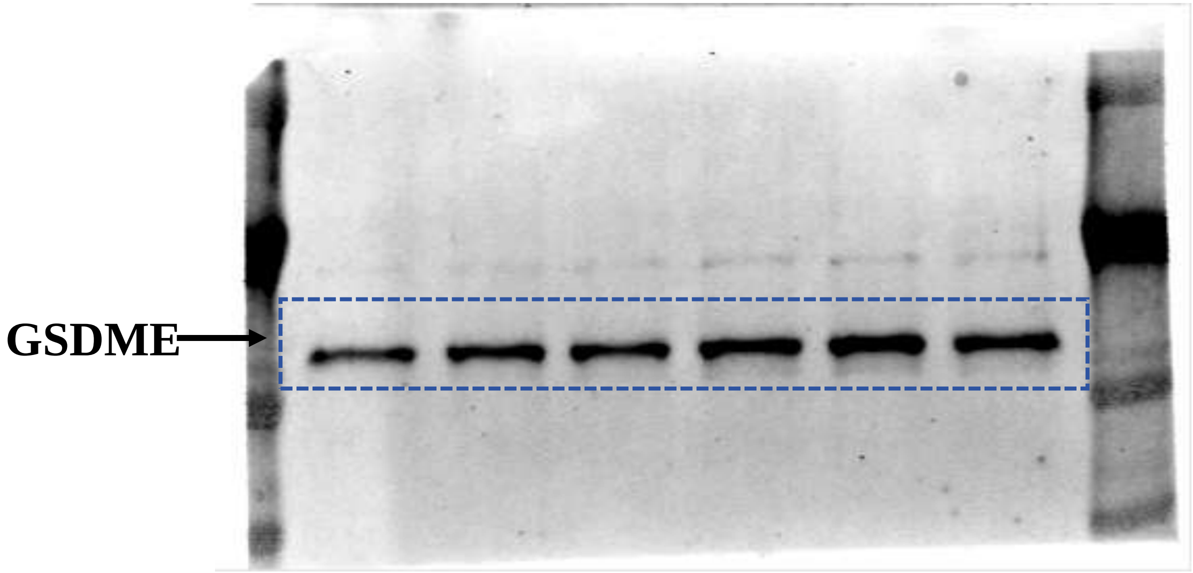

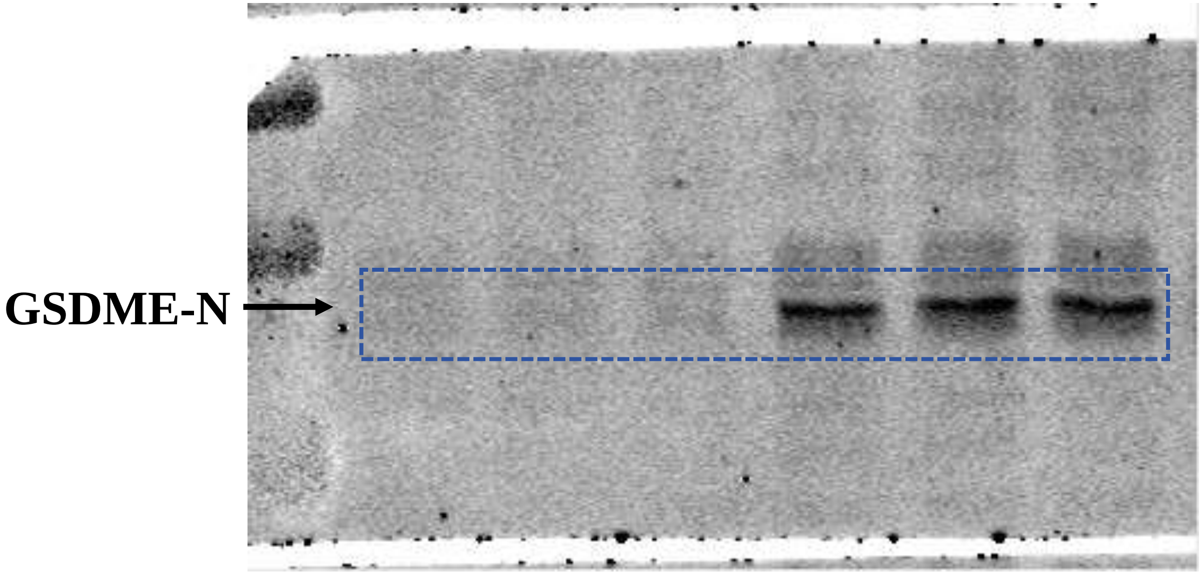

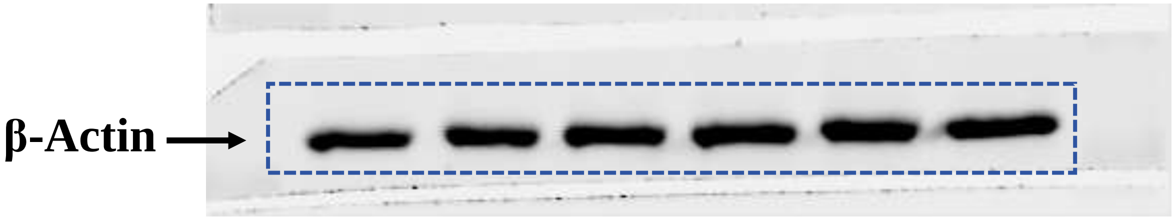

Full and uncropped western

Figure 5D

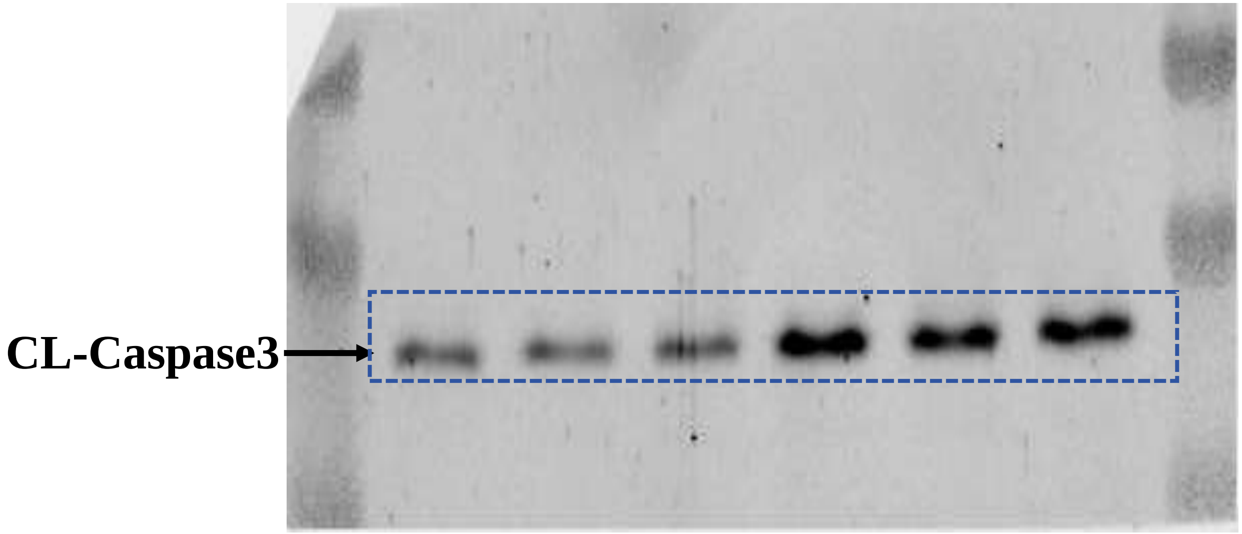

Figure 5F

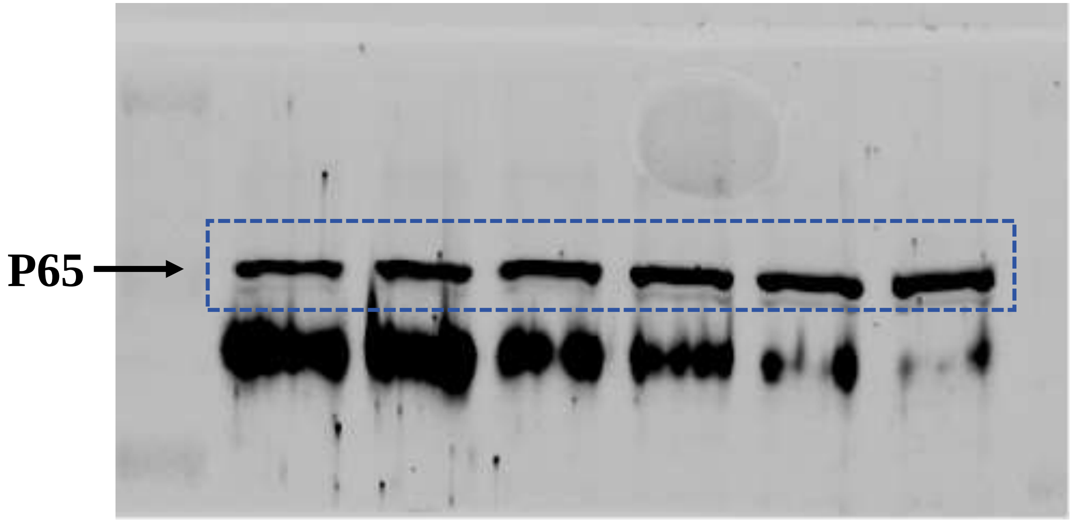

Figure 5F

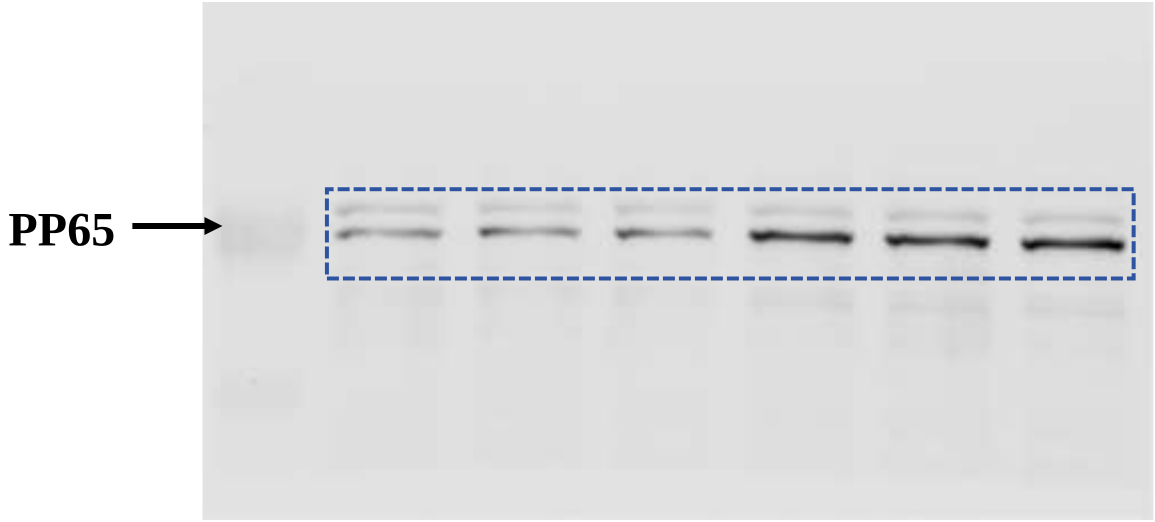

Figure 5F

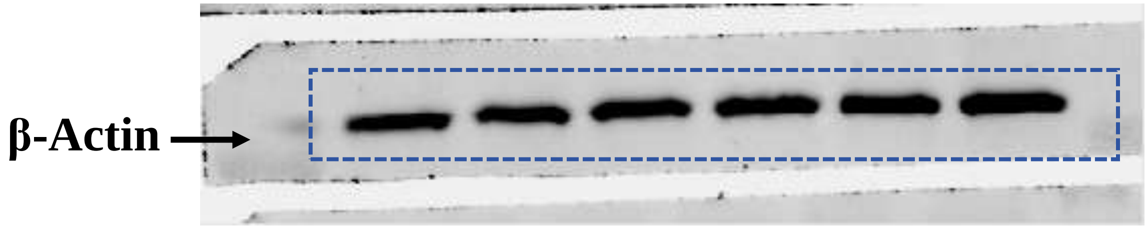

Full and uncropped western

Figure 6D

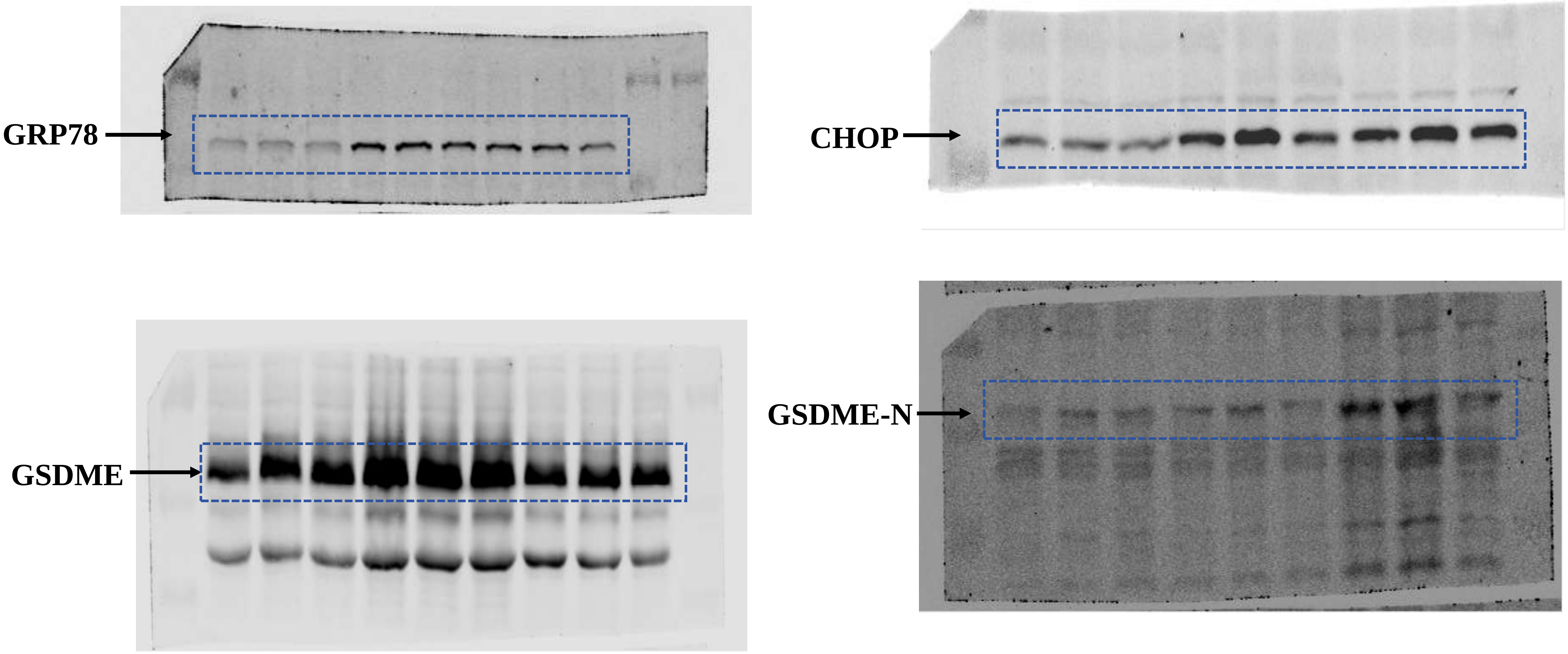

Full and uncropped western

Figure 6D

Caspase3

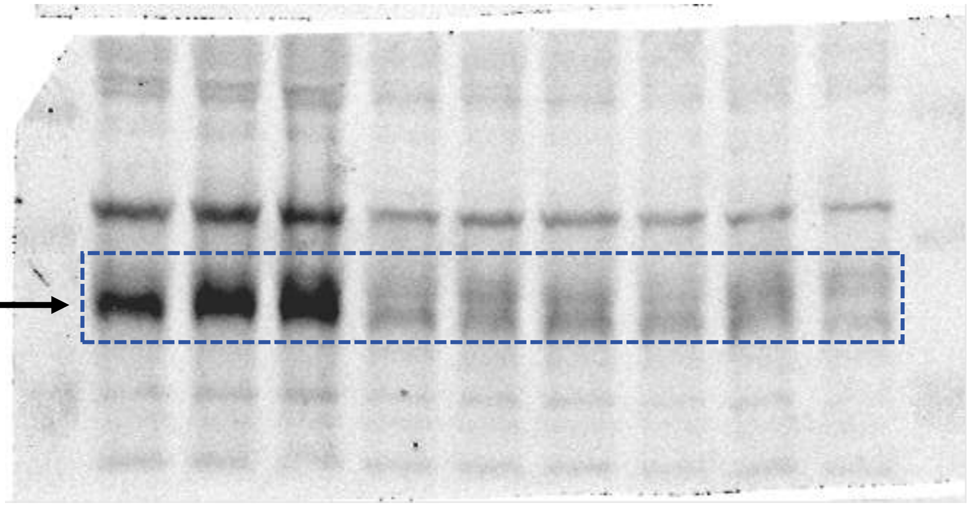

β-Actin

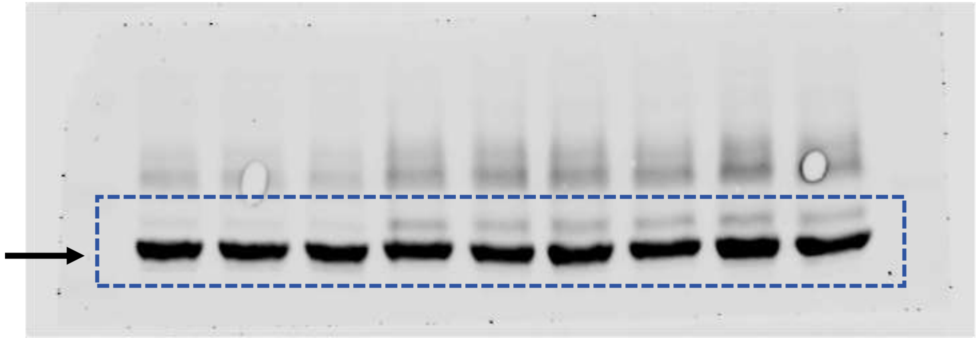

CL-Caspase3

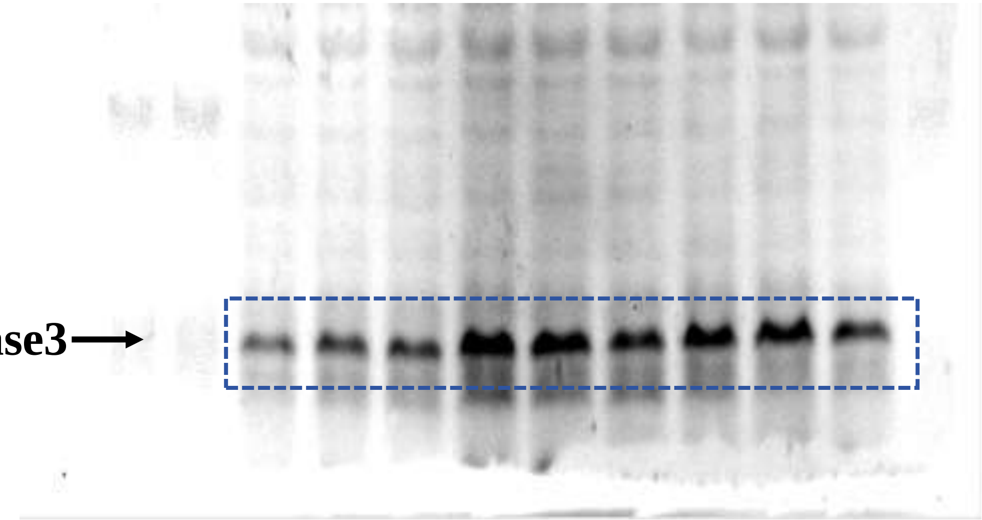

Full and uncropped western

Figure 6G

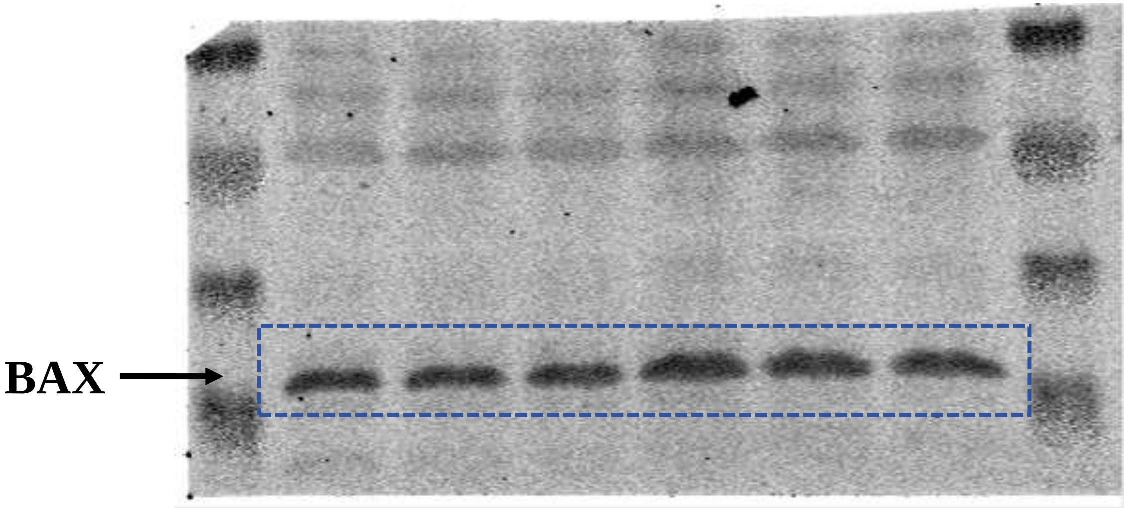

BAX

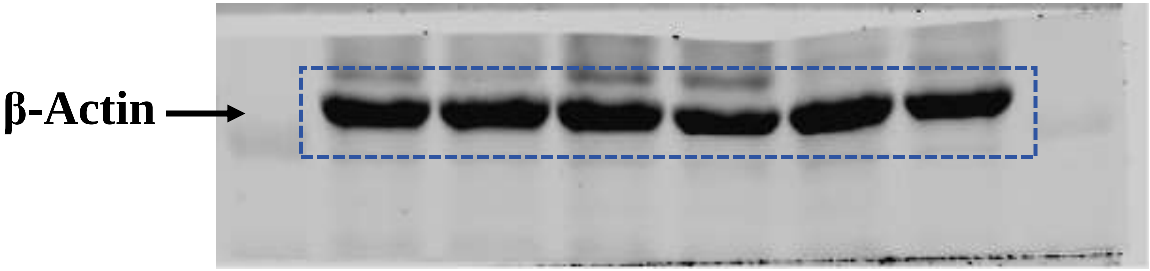

β-Actin

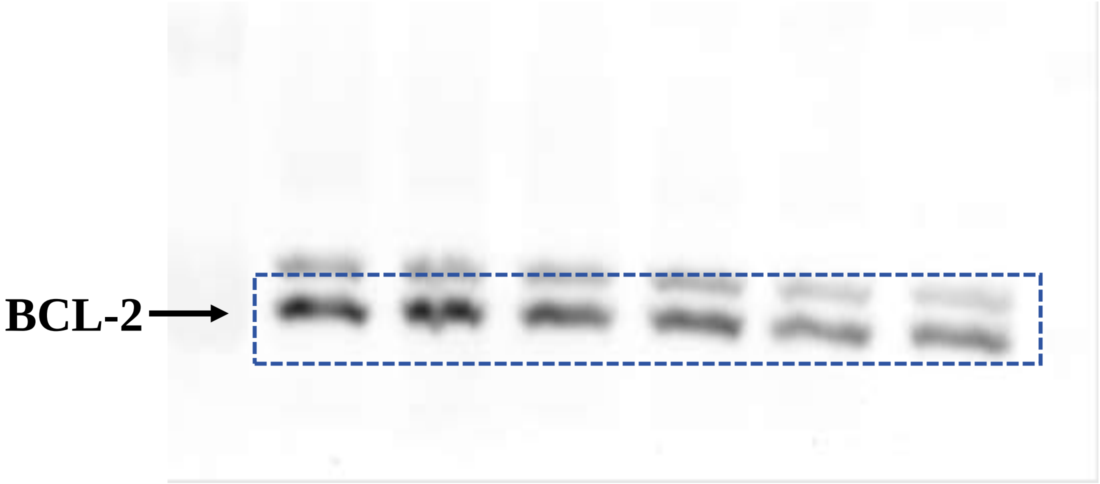

BCL-2

Full and uncropped western

Figure 6J

GRP78

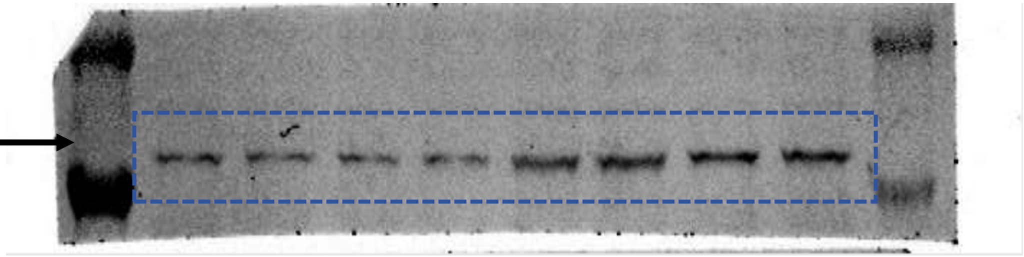

β-Actin

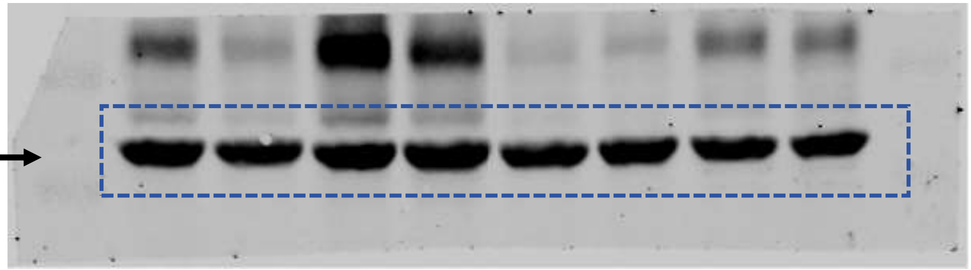

CHOP

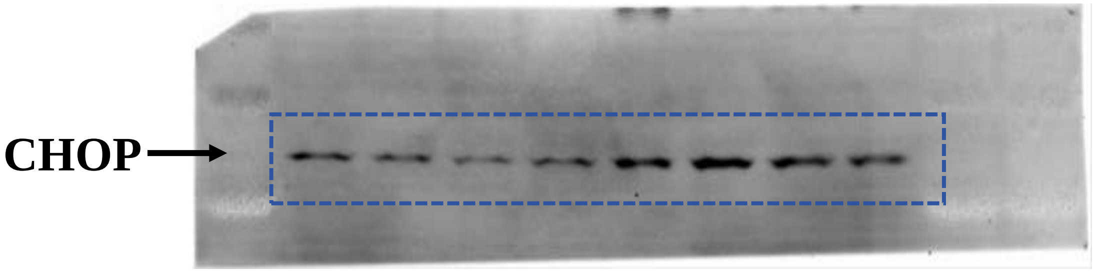

Full and uncropped western

Figure 7C

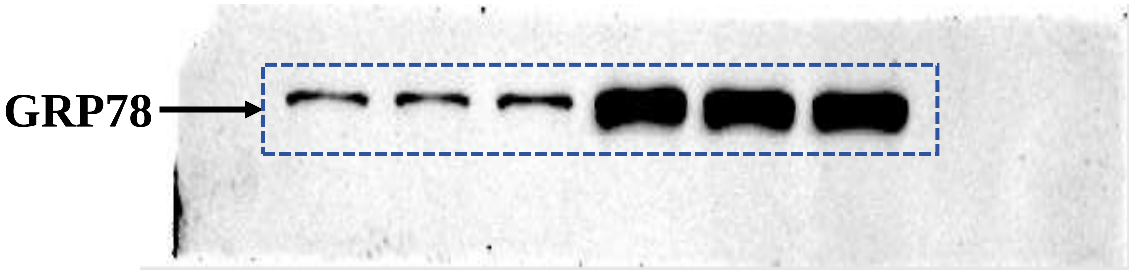

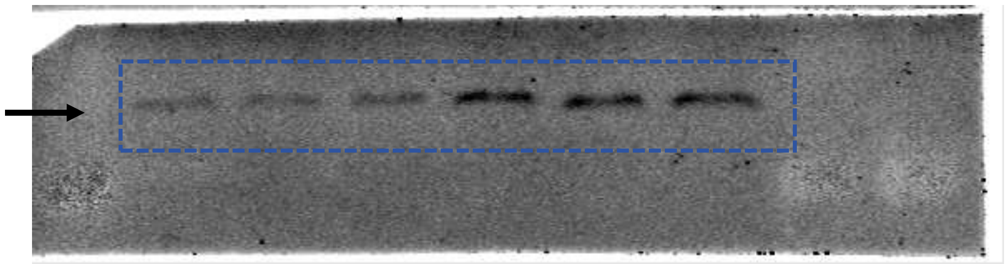

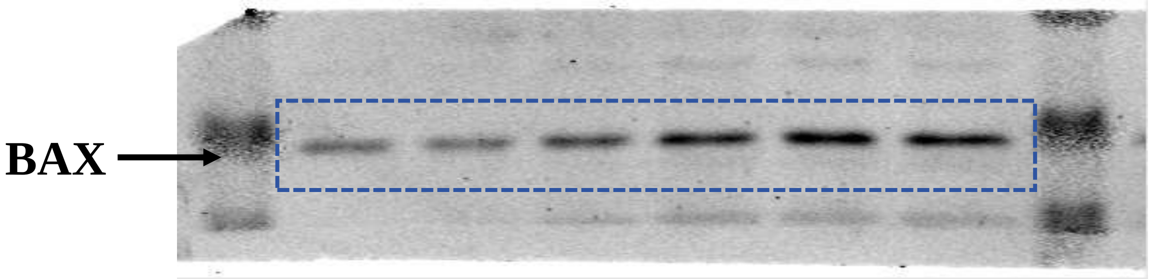

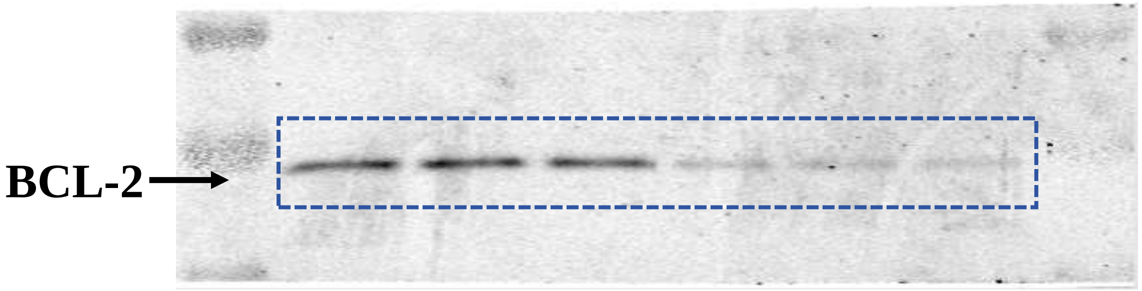

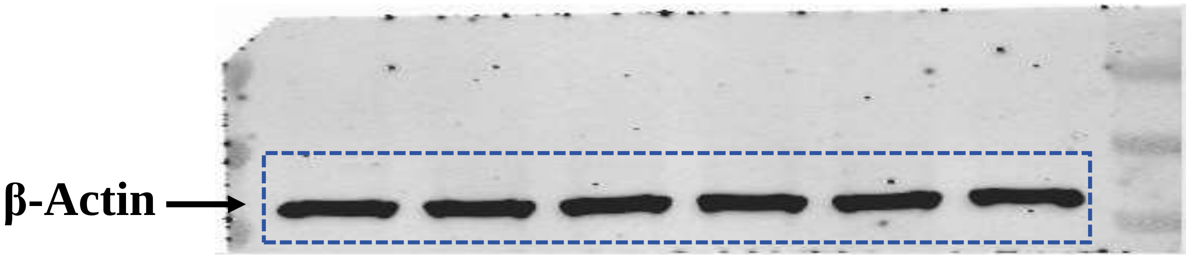

Full and uncropped western

Figure 7F

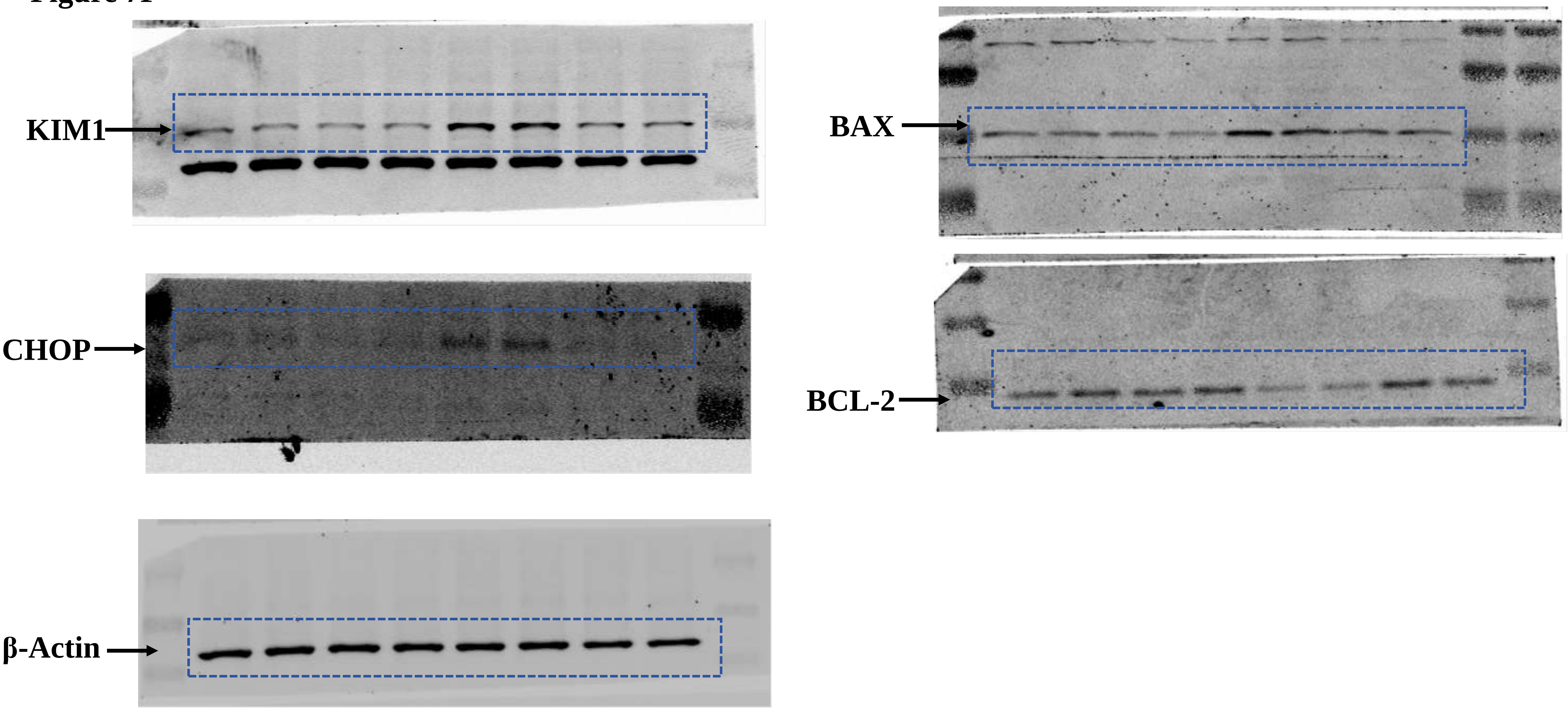

Full and uncropped western

Figure 8A

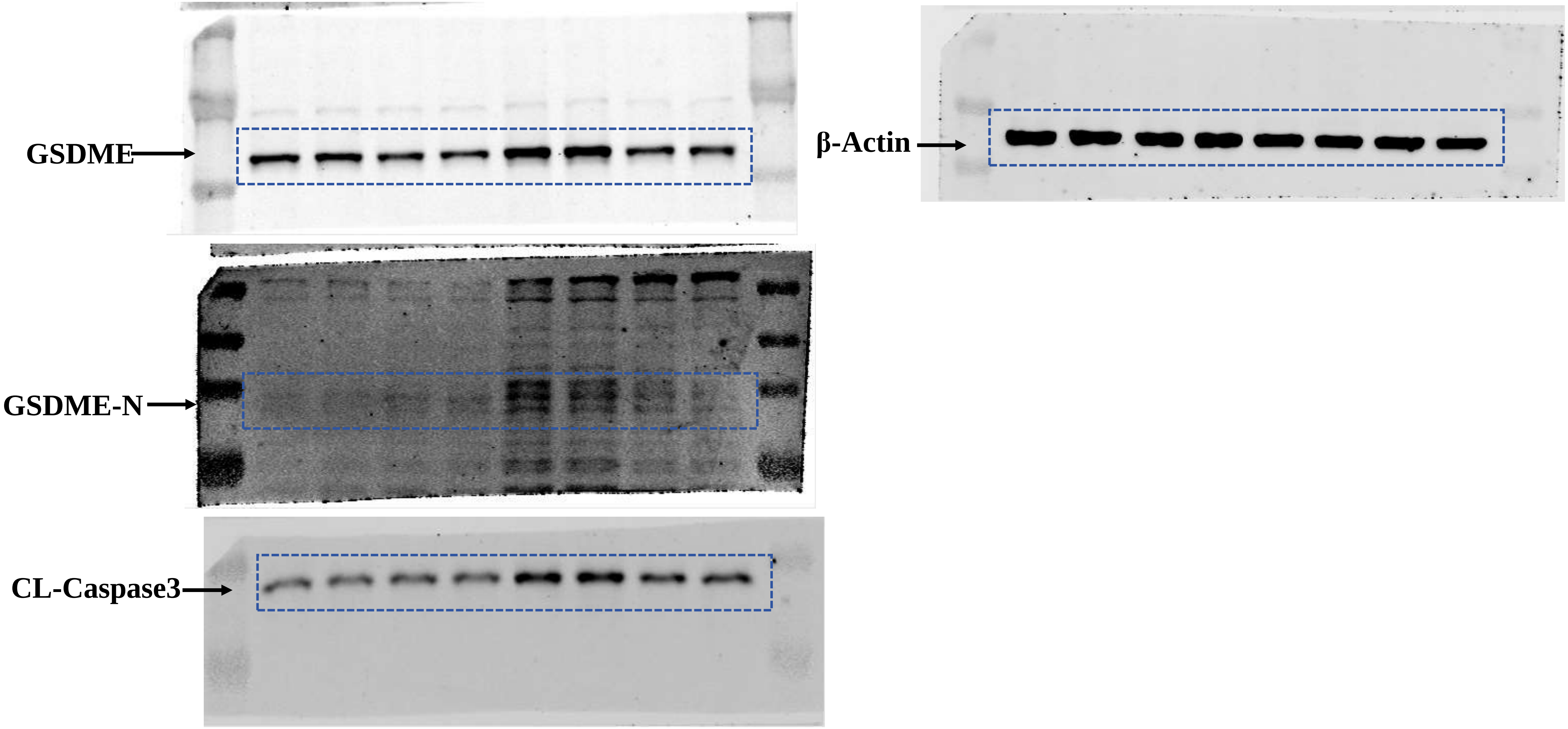

Full and uncropped western

Figure 8G

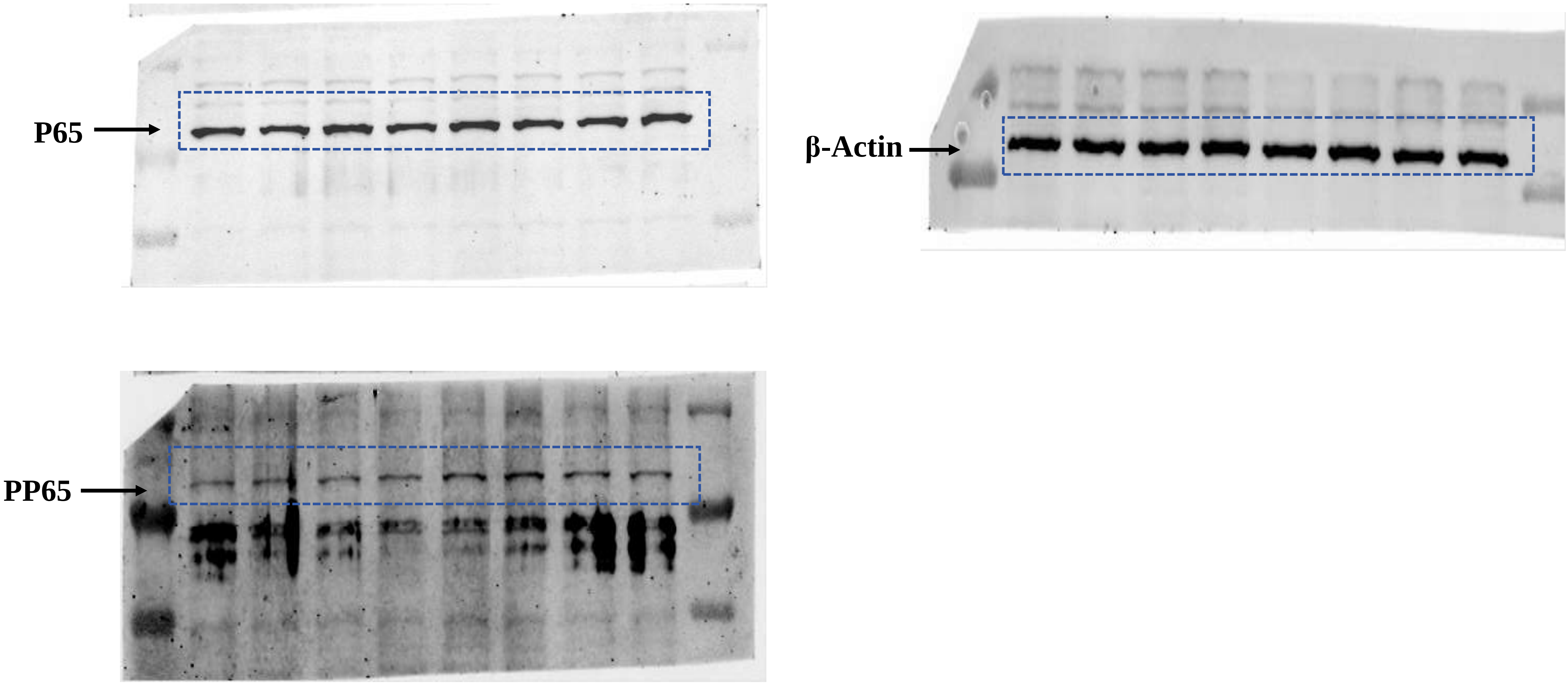

Full and uncropped western

Supplementary Fig. 1A

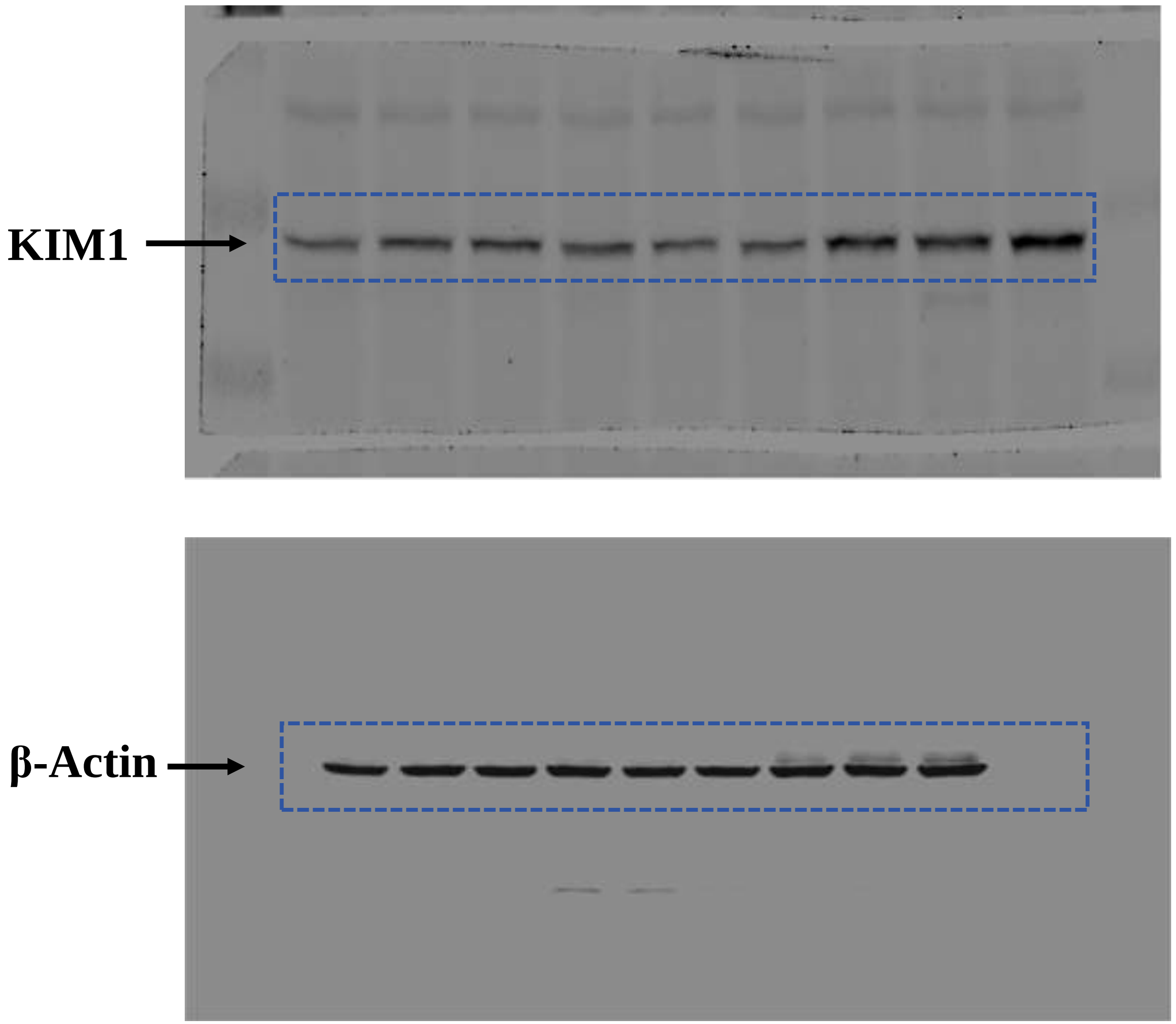

Full and uncropped western

Supplementary Fig. 2C

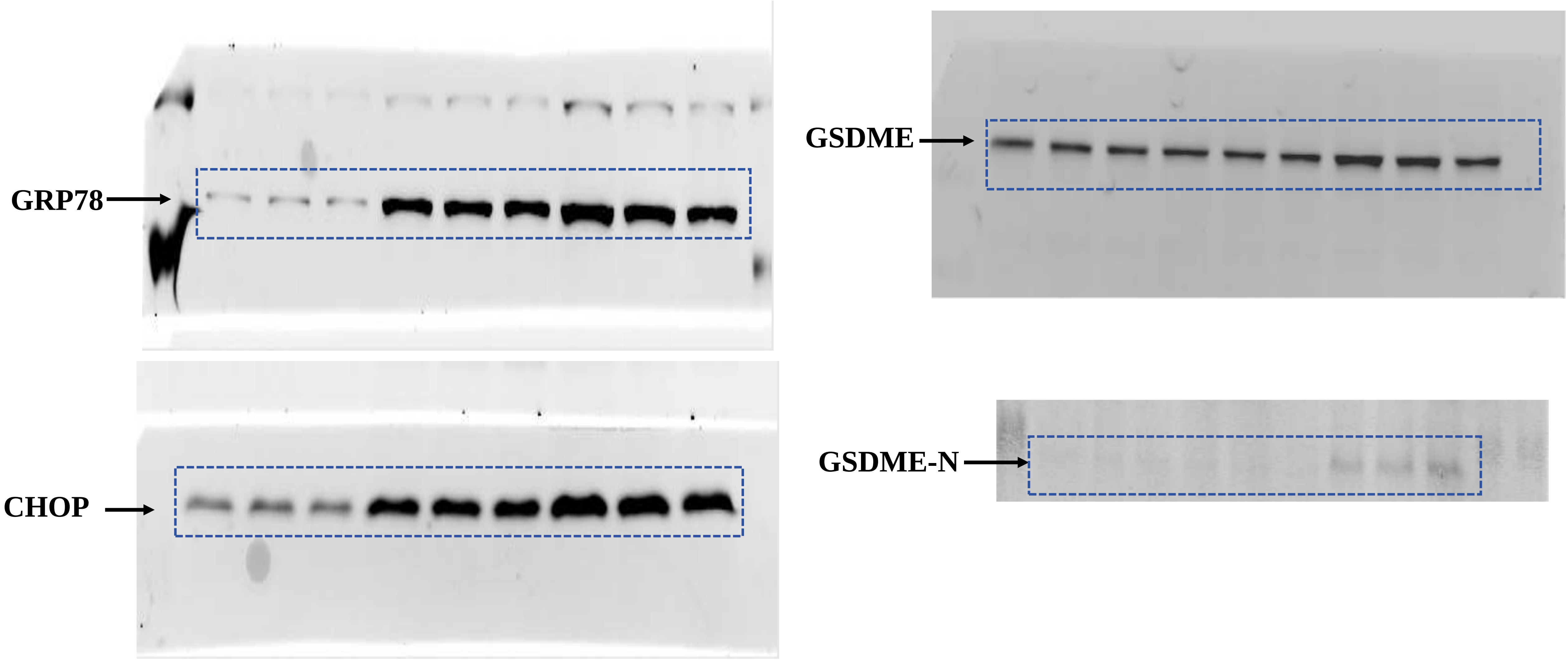

Full and uncropped western

Supplementary Fig. 2C

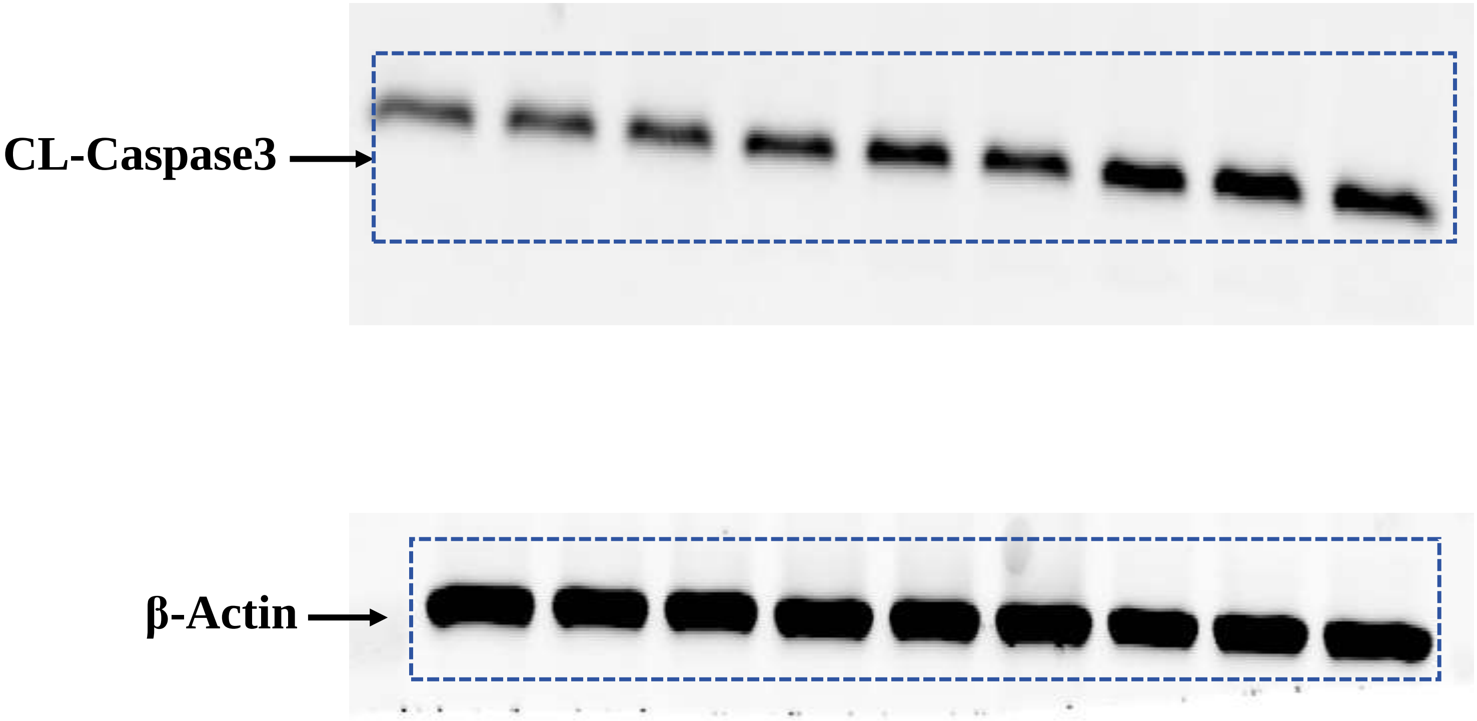

Supplement: Supplementary file 2 — Original Data File [file 41419_2024_6525_MOESM2_ESM.pdf]
