# Supplementary material for: CHOP-mediated Gasdermin E expression promotes pyroptosis, inflammation, and mitochondrial damage in renal ischemia-reperfusion injury
Source: Cell Death Dis. 2024 Feb 22;15(2):163. doi: 10.1038/s41419-024-06525-9 (PMC10883957; doi:10.1038/s41419-024-06525-9)
Supplement: Supplementary file 3 — Reporting Summary [file 41419_2024_6525_MOESM3_ESM.pdf]

SPRINGER NATURE

Corresponding Author Name: Tao Zhang
Manuscript Number: CDDIS-23-3850R

Reporting Summary

Springer Nature wishes to improve the reproducibility of the work that we publish. This checklist is used to ensure good reporting standards and to improve the reproducibility. Please respond completely to all questions relevant to your manuscript. For more information, please read the journal's Guide to Authors.

☑ **Check here to confirm that the following information is available in the Material & Methods section:**

- **The exact sample size (*n*)** for each experimental group/condition, given as a number, not a range
- **A description of the sample collection** allowing the reader to understand whether the samples represent technical or biological replicates (including how many animals, litters, culture, etc.)
- **A statement of how many times the experiment shown was replicated in the laboratory**
- **Definitions of statistical methods and measures**: For small sample sizes (n<5) descriptive statistics are not appropriate, instead plot individual data points
  - Very common tests, such as *t*-test, simple χ^2 tests, Wilcoxon and Mann-Whitney tests, can be unambiguously identified by name only, but more complex techniques should be described in the methods section
  - Are tests one-sided or two-sided?
  - Are there adjustments for multiple comparisons?
  - **Statistical test results**, e.g., ***P* values**
  - Definition of **'center values'** as **median or mean**;
  - Definition of **error bars as s.d. or s.e.m. or c.i.**

Please ensure that the answers to the following questions are CDDIS-23-3850 ***reported in the manuscript itself. We encourage you to include a specific subsection in the methods section for statistics, reagents and animal models. Below, provide the page number or section and paragraph number.***

Statistics and general methods

	**Reported in section/paragraph or page #**
1. How was the sample size chosen to ensure adequate power to detect a pre-specified effect size? (Give section/paragraph or page #)	us publications without prior power analysis. Exact numbers of animals used in individual.experiments are indicated in t he figure legends.Materials and Methods section, page 18, l ine 387- 400
For animal studies, include a statement about sample size estimate even if no statistical methods were used.	Sample size is estimate on the basis of our previous publicat ion without prior power analysis.Materials and Methods sec tion, page 18, line 387- 400
2. Describe inclusion/exclusion criteria if samples or animals were excluded from the analysis. Were the criteria pre-established? (Give section/paragraph or page #)	No data were excluded
3. If a method of randomization was used to determine how samples/animals were allocated to experimental groups and processed, describe it. (Give section/paragraph or page #)	All mice were age and sex-matched and then randomized in to the different groups.Materials and Methods section, pag e 18, line 387- 400
For animal studies, include a statement about randomization even if no randomization was used.	Animals are randomized based on the body weight.Material s and Methods section, page 18, line 387- 400

4. If the investigator was blinded to the group allocation during the experiment and/or when assessing the outcome, state the extent of blinding. (Give section/paragraph or page #)	The investigators were not blinded to group allocation during experiments. Conclusions were made based on quantitative parameters and statistical significance of the data, and thus on experimental observations, independent of blinding. Materials and Methods section, page 18, line 387- 400
For animal studies, include a statement about blinding even if no blinding was done.	The investigators were not blinded to group allocation during experiments.
5. For every figure, are statistical tests justified as appropriate?	Yes, statistical tests are justified as appropriate for every Figure.
Do the data meet the assumptions of the tests (e.g., normal distribution)?	Yes
Is there an estimate of variation within each group of data?	Yes
Is the variance similar between the groups that are being statistically compared? (Give section/paragraph or page #)	Given that we used non parametric test to compare the means within the groups, it does not require estimation of the variance.

Reagents

	**Reported in section/paragraph or page #**
6. Report the source of antibodies (vendor and catalog number)	2895, CST, 1:1000),phospho-NF-κB p65 (3033, CST, 1:1000), NF-κB p65 (8242, CST, 1:1000), IRDyeTM800-conjugated secondary antibody (Rockland Immunochemicals).
7. Identify the source of cell lines and report if they were recently authenticated (e.g., by STR profiling) and tested for mycoplasma contamination	The American Type Culture Collection (ATCC, USA) sold the human renal proximal tubular epithelial cell line (HK-2) that was used in this study. Each batch of HK2 is sterile and tested negative for mycoplasma, bacteria, yeast and fungi according to the manufacturer.

Animal Models

	**Reported in section/paragraph or page #**
8. Report species, strain, sex and age of animals	nces donated GSDME-deficient (GSDME-/-, knockout) C57BL/6J mice.
9. For experiments involving live vertebrates, include a statement of compliance with ethical regulations and identify the committee(s) approving the experiments.	All the related studies were authorised by the Biomedical Ethics Committee of Anhui Medical University (Ethical Approval No. LLSC20190660) and were implemented with the subjects written consent and understanding.

10. We recommend consulting the ARRIVE guidelines (*PLoS Biol.* **8**(6), e1000412,2010) to ensure that other relevant aspects of animal studies are adequately reported.

Human subjects

Reported in section/paragraph or page #

11. Identify the committee(s) approving the study protocol.	Materials and Methods section, Page 18 Line 380 -386
12. Include a statement confirming that informed consent was obtained from all subjects.	Materials and Methods section, page 18, line 380 -386
13. For publication of patient photos, include a statement confirming that consent to publish was obtained.	Not applicable
14. Report the clinical trial registration number (at ClinicalTrials.gov or equivalent).	Not applicable

15. For phase II and III randomized controlled trials, please refer to the CONSORT statement and submit the CONSORT checklist with your submission.

16. For tumor marker prognostic studies, we recommend that you follow the REMARK reporting guidelines.

Data deposition

Reported in section/paragraph or page #

17. Provide accession codes for deposited data. Data deposition in a public repository is mandatory for:<br>a. Protein, DNA and RNA sequences<br>b. Macromolecular structures<br>c. Crystallographic data for small molecules<br>d. Microarray data	Not applicable

Deposition is strongly recommended for many other datasets for which structured public repositories exist; more details on our data policy are available in the Guide to Authors. We encourage the provision of other source data in supplementary information or in unstructured repositories such as Figshare and Dryad. We encourage publication of Data Descriptors (see Scientific Data) to maximize data reuse.

18. If computer code was used to generate results that are central to the paper's conclusions, include a statement in the Methods section under "**Code availability**" to indicate whether and how the code can be accessed. Include version information as necessary and any restrictions on availability.	Not applicable
